# Proteomic profiling of low muscle and high fat mass: a machine learning approach in the KORA S4/FF4 study

**DOI:** 10.1002/jcsm.12733

**Published:** 2021-06-20

**Authors:** Marie‐Theres Huemer, Alina Bauer, Agnese Petrera, Markus Scholz, Stefanie M. Hauck, Michael Drey, Annette Peters, Barbara Thorand

**Affiliations:** ^1^ Institute of Epidemiology Helmholtz Zentrum München, German Research Center for Environmental Health (GmbH) Neuherberg Germany; ^2^ Research Unit Protein Science Helmholtz Zentrum München, German Research Center for Environmental Health (GmbH) Neuherberg Germany; ^3^ Institute for Medical Informatics, Statistics and Epidemiology (IMISE) Universität Leipzig Leipzig Germany; ^4^ Medizinische Klinik und Poliklinik IV, Schwerpunkt Akutgeriatrie Klinikum der Universität München (LMU) Munich Germany; ^5^ German Center for Diabetes Research (DZD) München‐Neuherberg Germany; ^6^ Chair of Epidemiology, Institute for Medical Information Processing, Biometry and Epidemiology, Medical Faculty Ludwig‐Maximilians‐Universität München Munich Germany

**Keywords:** Appendicular skeletal muscle mass, Body fat mass index, Fat mass, Muscle mass, Machine learning, Proteomics

## Abstract

**Background:**

The coexistence of low muscle mass and high fat mass, two interrelated conditions strongly associated with declining health status, has been characterized by only a few protein biomarkers. High‐throughput proteomics enable concurrent measurement of numerous proteins, facilitating the discovery of potentially new biomarkers.

**Methods:**

Data derived from the prospective population‐based Cooperative Health Research in the Region of Augsburg S4/FF4 cohort study (median follow‐up time: 13.5 years) included 1478 participants (756 men and 722 women) aged 55–74 years in the cross‐sectional and 608 participants (315 men and 293 women) in the longitudinal analysis. Appendicular skeletal muscle mass (ASMM) and body fat mass index (BFMI) were determined through bioelectrical impedance analysis at baseline and follow‐up. At baseline, 233 plasma proteins were measured using proximity extension assay. We implemented boosting with stability selection to enable false positives‐controlled variable selection to identify new protein biomarkers of low muscle mass, high fat mass, and their combination. We evaluated prediction models developed based on group least absolute shrinkage and selection operator (lasso) with 100× bootstrapping by cross‐validated area under the curve (AUC) to investigate if proteins increase the prediction accuracy on top of classical risk factors.

**Results:**

In the cross‐sectional analysis, we identified kallikrein‐6, C‐C motif chemokine 28 (CCL28), and tissue factor pathway inhibitor as previously unknown biomarkers for muscle mass [association with low ASMM: odds ratio (OR) per 1‐SD increase in log2 normalized protein expression values (95% confidence interval (CI)): 1.63 (1.37–1.95), 1.31 (1.14–1.51), 1.24 (1.06–1.45), respectively] and serine protease 27 for fat mass [association with high BFMI: OR (95% CI): 0.73 (0.61–0.86)]. CCL28 and metalloproteinase inhibitor 4 (TIMP4) constituted new biomarkers for the combination of low muscle and high fat mass [association with low ASMM combined with high BFMI: OR (95% CI): 1.32 (1.08–1.61), 1.28 (1.03–1.59), respectively]. Including protein biomarkers selected in ≥90% of group lasso bootstrap iterations on top of classical risk factors improved the performance of models predicting low ASMM, high BFMI, and their combination [delta AUC (95% CI): 0.16 (0.13–0.20), 0.22 (0.18–0.25), 0.12 (0.08–0.17), respectively]. In the longitudinal analysis, N‐terminal prohormone brain natriuretic peptide (NT‐proBNP) was the only protein selected for loss in ASMM and loss in ASMM combined with gain in BFMI over 14 years [OR (95% CI): 1.40 (1.10–1.77), 1.60 (1.15–2.24), respectively].

**Conclusions:**

Proteomic profiling revealed CCL28 and TIMP4 as new biomarkers of low muscle mass combined with high fat mass and NT‐proBNP as a key biomarker of loss in muscle mass combined with gain in fat mass. Proteomics enable us to accelerate biomarker discoveries in muscle research.

## Introduction

For several decades, the disorder of low muscle mass was not recognized as a severe condition, although it is associated with various pathological conditions such as non‐alcoholic fatty liver disease, type 2 diabetes,^1^ hypertension,^2^ and cardiovascular mortality.^3^ A milestone turn in perception of condition severity constituted the assignment of an ICD‐10‐CM code for the term sarcopenia in 2016. Besides the increasing awareness of low muscle mass, early decisive research observed that the combination of low muscle and high fat mass had a more detrimental effect on disability in daily living activity,^4^ multi‐morbidity,^5^ and an increased 10 year cardiovascular disease (CVD) risk^6^ in comparison with participants solely experiencing low muscle mass.

Previous studies investigated the association of different muscle mass and fat mass parameters with a low number of biomarkers. Most studies focused on classical inflammatory biomarkers, predominantly C‐reactive protein (CRP) and interleukin (IL)‐6, and found contradictory results regarding the relation to muscle mass (fat free mass index^7^ and loss of appendicular skeletal muscle mass (ASMM)^8^, ^9^). The combination of low muscle mass (appendicular lean mass) and high body fat has been investigated and observed to be independently associated with CRP and fibrinogen.^10^ However, there has been the concern that a low number of biomarkers might be insufficient in describing disease development. The principle of parsimony, that is, only selecting a small set of biomarkers as predictors for the outcome, could provide incomplete results as few biomarkers only reflect the most prominent proteins related to general processes.^11^ As a response, multiplex measurements including a high number of proteins, that is, proteomics, have been established over the last years. Several recently published cross‐sectional and longitudinal studies used different proteomics measurements to investigate various body composition parameters including body mass index (BMI), waist circumference (WC), waist‐to‐hip ratio (WHR), body fat mass (kg), and body fat (%),^12^, ^13^, ^14^, ^15^, ^16^, ^17^, ^18^, ^19^ but only one study investigated a muscle mass parameter, lean body mass (kg).^17^ Studies using high‐throughput proteomics to assess associations with muscle and fat mass parameters in combination are lacking. The aim of this study is to identify new protein biomarkers of low muscle, high fat mass, and their combination as well as their changes over a 14 year follow‐up period.

## Methods

### Study population

The analysis is based on data from the population‐based Cooperative Health Research in the Region of Augsburg (KORA) study, conducted in Southern Germany. 4261 individuals participated in the KORA S4 baseline examinations,^20^ and 2279 additionally participated in the second follow‐up study KORA FF4 (2013–2014).

The present analysis was restricted to participants aged 55–74 years at baseline (*n* = 1653), who were invited to the study centre after an overnight fast of at least 8 h. After exclusions, the cross‐sectional analysis included 1478 participants (756 men and 722 women) of which 1315 participants complied with the overnight fasting and 163 participants did not. Out of these 1478 participants, 608 participants (315 men and 293 women) with a median follow‐up time of 13.5 years (25th percentile: 13.5 years, 75th percentile: 13.6 years) remained for the longitudinal analysis. Exclusion criteria of the cross‐sectional and longitudinal analysis are illustrated in Supporting information, *Figure* S1.

At the S4 and FF4 surveys, all participants were examined by trained medical personnel. In the S4 survey, sociodemographic data, lifestyle, medical history, and medication use were assessed in a standardized face‐to‐face interview.^20^

### Exposure

Plasma samples collected at S4 in 1999–2001 were used to measure CVD‐ and inflammation‐related protein biomarkers. Protein measurements were performed using proximity extension assay (PEA) technology developed by Olink® (Olink Proteomics, Uppsala, Sweden) with the three panels Olink® Multiplex CVDII, CVDIII, and Inflammation, each comprising 92 protein biomarkers. Details regarding measurement protocol are described elsewhere.^21^ The Olink® platform provided the protein biomarkers as log2‐normalized protein expression (NPX) values. We further divided the values by their respective standard deviation using the total study population with available data before exclusions. After exclusions, 233 protein biomarkers remained for the present analysis. Exclusion criteria are described in Supporting information, *Figure* S2. Supporting information, *Tables* S1–S3 provide detailed information regarding all 276 measured biomarkers of the three panels before exclusions.

### Outcome

The parameters requisite to calculate the continuous outcome variables ASMM in kilogram and body fat mass index (BFMI) in kilograms per square meter were assessed at S4 and FF4 using bioelectrical impedance analysis (BIA) with the BIA 2000‐S (DATA‐INPUT GmbH, Frankfurt, Germany). The calculations are included in the Supporting information. The binary outcomes included the risk group low ASMM, representing the 25% (*n* = 370) of participants with the lowest ASMM and its corresponding control group, the remaining 75% (*n* = 1108). The risk group of the outcome high BFMI included the 25% (*n* = 370) of participants with the highest BFMI and its corresponding control group, the remaining 75% (*n* = 1108). Sex‐specific cut points were used for this purpose. The risk group for the combined outcome of low ASMM and high BFMI was determined by intersecting the 40% of participants with the lowest ASMM and the 40% of participants with the highest BFMI. This group consists of 7% (*n* = 110) of the total study population and the corresponding control group of the remaining 93% (*n* = 1368). Cut points of 40% were chosen for this outcome to ensure a sufficiently large size of the risk group while preserving a relatively extreme value of low ASMM and high BFMI. Currently, no standardized definition for the combination of both outcomes exists for European populations. For the longitudinal analysis, we used the changes of ASMM and BFMI between baseline and follow‐up relative to baseline. Therefore, we changed the variables' descriptions from ‘low ASMM’ to ‘strong decrease in ASMM’ and ‘high BFMI’ to ‘strong increase in BFMI’, while the cut points based on the percentages remained the same. Detailed descriptions of the cross‐sectional and longitudinal outcomes are included in Supporting information, *Table* S4, *Figure* S3, and *Table* S5.

### Covariates

The covariates (association analysis)/classical risk factors (prediction analysis) included age, high‐density lipoprotein, triglycerides, glycated haemoglobin, estimated glomerular filtration rate (eGFR), albumin (all continuous), sex (female/male), physical activity (high/moderate/low/no activity), hypertension (no/yes), smoking status (never/former/current smoker), education (>10 years/≤10 years), alcohol intake [0 g/day, 0.1–39.9 g/day (men)/0.1–19.9 g/day (women), ≥40 g/day [men]/≥20 g/day (women)], and intake of lipid‐lowering medication (no/yes). Detailed information describing their measurements are available in the Supporting information.

### Statistical analysis

**Figure 1:**
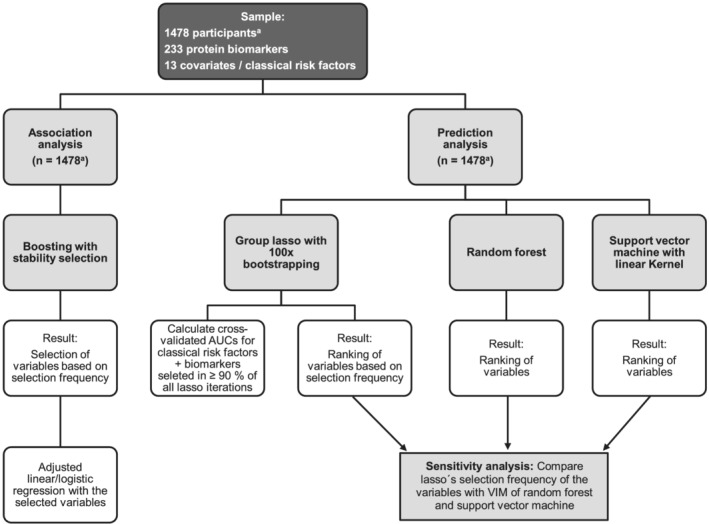
Statistical analysis plan. AUC, area under the curve; lasso, least absolute shrinkage and selection operator; VIM, variable importance measure. ^a^1478 participants in the cross‐sectional analysis; 608 participants in the longitudinal analysis.

Test results with two‐sided *P* value <0.05 were considered statistically significant. Analysis workflow is depicted in *Figure* 1 and described in the Supporting information. We implemented the same statistical approach in both, cross‐sectional and longitudinal analyses.

We separated the analysis into two parts to investigate two different analysis goals, ‘association’ and ‘prediction’. As both terms have various applications, in the following, we specify this paper's meaning of the terms. The goal of the association analysis comprised the accurate selection of biomarkers associated with the outcomes independent of covariates. Therefore, we implemented boosting with stability selection as it allows finite error control of false positives enabling accurate variable selection. The paper validating this method explains that its prediction accuracy can suffer as the true positive rate is due to a tight error control usually lower compared with prediction methods without stability selection. ‘Prediction and variable selection are two different goals.’^22^ Our goal of the prediction analysis was to identify biomarker models able to predict unknown data using methods with a high predictive accuracy. The sensitivity analysis was employed to compare the highest ranked variables between these methods.

## Results

Participant characteristics of the analysed population are listed in Supporting information, *Tables* S6 and S7. Partial correlation analysis between ASMM and BFMI adjusted for age and sex resulted in a Spearman rank correlation coefficient of 0.57. Coefficients to other body composition parameters constituted for BFMI and BMI 0.93, ASMM and BMI 0.68, BFMI and WC 0.84, ASMM and WC 0.68, BFMI and WHR 0.52, and ASMM and WHR 0.30.

### Cross‐sectional association of appendicular skeletal muscle mass/body fat mass index with protein biomarkers

**Figure 2:**
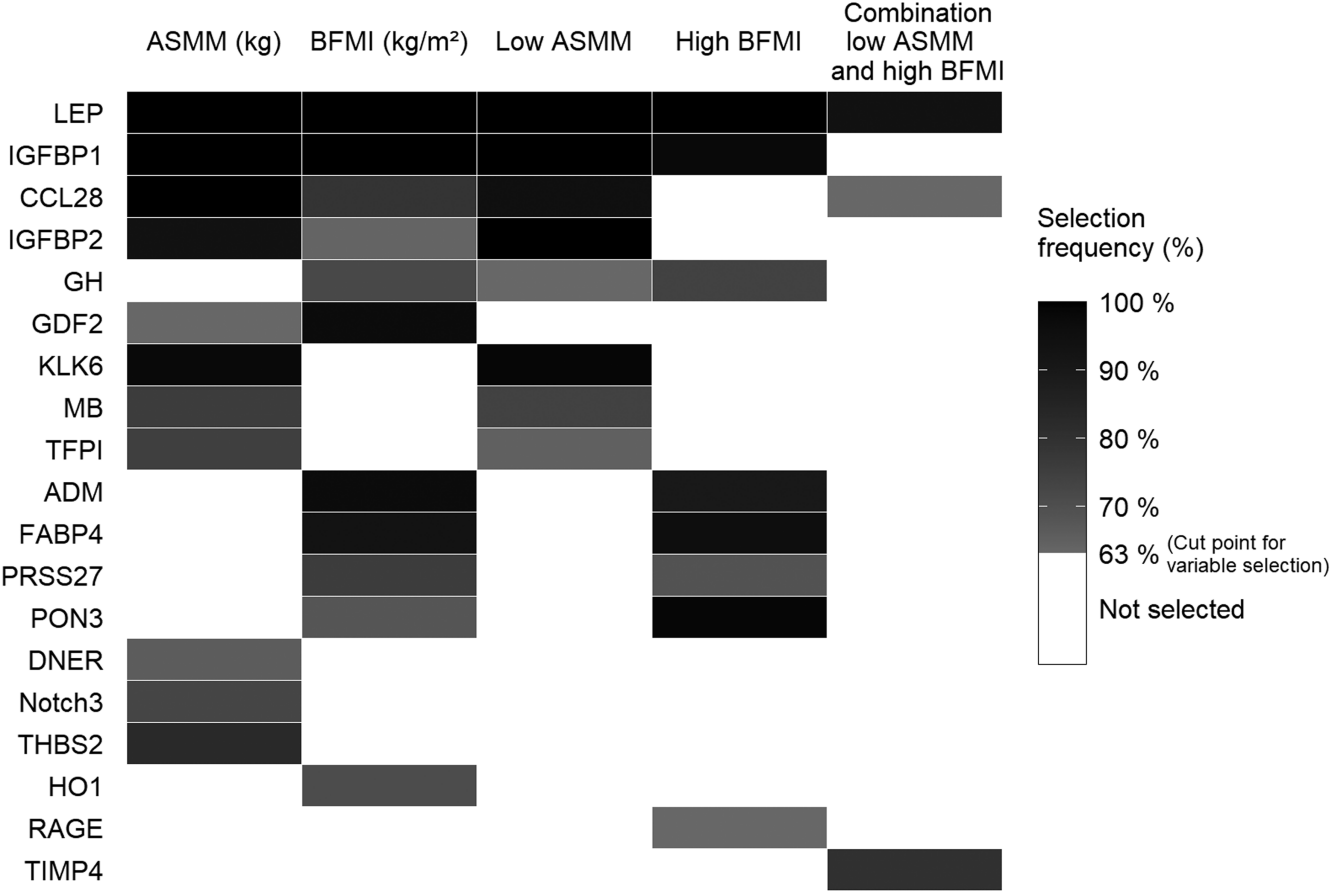
Association analysis — boosting with stability selection — comparison of protein biomarker selection between the outcomes. Protein biomarkers are primarily ordered according to the number of outcomes the biomarkers were selected for and secondary according to their selection for the outcomes in the table from left to right. Only protein biomarkers are included that were selected for at least one outcome. The cut point for variable selection was a selection frequency of 63%, which was determined by the algorithm based on the number of variables available for selection, the number of selected variables per iteration, and the maximum number of tolerable false positives. ASMM, appendicular skeletal muscle mass; BFMI, body fat mass index.

*Table* 1 displays the strength of associations of protein biomarkers selected by boosting with stability selection with the outcomes adjusted for Models 1 and 2. *Figure* 2 illustrates a comparison of the selected biomarkers between the outcomes.

**Table 1:** Association analysis — boosting with stability selection and regression analyses

Boosting with stability selection		Linear regression models			
Selected variables	Selection frequency	β (95% CI)	*P* value	β (95% CI)	*P* value
ASMM (kg)					
		Model 1		Model 2 (Model 1 + BFMI)	
IGFBP1	100%	**−0.38** (−0.51, −0.26)	**4.74e‐09**	**−0.24** (−0.35, −0.12)	**6.05e‐05**
LEP	100%	**0.69** (0.54, 0.84)	**<2e‐16**	**−0.22** (−0.39, −0.05)	**0.010667**
CCL28	100%	**−0.29**(−0.39, −0.18)	**6.37e‐08**	**−0.21** (−0.30, −0.11)	**1.80e‐05**
KLK6	98%	**−0.46** (−0.58, −0.33)	**3.41e‐12**	**−0.34** (−0.46, −0.23)	**5.95e‐09**
IGFBP2	94%	**−0.25** (−0.39, −0.10)	**0.000764**	**−0.22** (−0.35, −0.09)	**0.000843**
THBS2	83%	**0.18** (0.06, 0.29)	**0.003159**	0.08 (−0.03, 0.19)	0.142810
MB	76%	**0.28** (0.16, 0.41)	**1.25e‐05**	**0.20** (0.08, 0.31)	**0.000690**
TFPI	75%	**−0.21** (−0.33, −0.10)	**0.000147**	**−0.22** (−0.32, −0.12)	**2.40e‐05**
Notch3	73%	**0.52** (0.37, 0.66)	**7.72e‐12**	**0.43** (0.30, 0.57)	**1.84e‐10**
DNER	66%	**−0.19** (−0.31, −0.08)	**0.000910**	**−0.12** (−0.23, −0.02)	**0.021671**
GDF2	63%	**−0.21** (−0.34, −0.09)	**0.000496**	−0.07 (−0.18, 0.04)	0.212388

BFMI (kg/m^2^)					
		Model 1		Model 2 (Model 1 + ASMM)	
IGFBP1	100%	**−0.36** (−0.47, −0.24)	**1.46e‐09**	**−0.23** (−0.33, −0.12)	**1.74e‐05**
LEP	100%	**1.32** (1.15, 1.49)	**<2e‐16**	**1.09** (0.93, 1.24)	**<2e‐16**
GDF2	97%	**−0.26** (−0.37, −0.16)	**1.42e‐06**	**−0.20** (−0.30, −0.11)	**2.32e‐05**
ADM	97%	**0.30** (0.16, 0.43)	**2.45e‐05**	**0.25** (0.13, 0.37)	**7.27e‐05**
FABP4	93%	**0.78** (0.62, 0.93)	**<2e‐16**	**0.70** (0.56, 0.83)	**<2e‐16**
CCL28	79%	**−0.17** (−0.26, −0.07)	**0.000425**	−0.05 (−0.13, 0.03)	0.257357
PRSS27	76%	**−0.17** (−0.27, −0.08)	**0.000399**	**−0.12** (−0.21, −0.03)	**0.005940**
GH	72%	**−0.13** (−0.24, −0.03)	**0.013998**	**−0.12** (−0.22, −0.03)	**0.012302**
HO1	71%	0.08 (−0.02, 0.18)	0.124910	0.03 (−0.05, 0.12)	0.444350
PON3	68%	**−0.27** (−0.39, −0.16)	**4.55e‐06**	**−0.20** (−0.30, −0.09)	**0.000206**
IGFBP2	64%	**−0.18** (−0.31, −0.04)	**0.010195**	−0.08 (−0.20, 0.04)	0.181302

		Logistic regression models			
Selected variables	Selection frequency	OR (95% CI)	*P* value	OR (95% CI)	*P* value
Low ASMM					
		Model 1		Model 2 (Model 1 + high BFMI)	
IGFBP2	100%	1.14 (0.94, 1.39)	0.180094	1.14 (0.93, 1.39)	0.207678
IGFBP1	100%	**1.45** (1.19, 1.75)	**0.000166**	**1.40** (1.15, 1.70)	**0.000844**
LEP	100%	**0.51** (0.42, 0.64)	**6.21e‐10**	**0.67** (0.54, 0.84)	**0.000537**
KLK6	99%	**1.63** (1.37, 1.95)	**3.80e‐08**	**1.61** (1.35, 1.93)	**1.50e‐07**
CCL28	95%	**1.31** (1.14, 1.51)	**0.000177**	**1.29** (1.12, 1.49)	**0.000590**
MB	74%	**0.62** (0.52, 0.75)	**2.88e‐07**	**0.64** (0.53, 0.77)	**3.03e‐06**
TFPI	65%	**1.24** (1.06, 1.45)	**0.006704**	**1.23** (1.05, 1.45)	**0.008969**
GH	63%	**1.18** (1.00, 1.39)	**0.048414**	1.16 (0.98, 1.37)	0.083713

High BFMI					
		Model 1		Model 2 (Model 1 + low ASMM)	
LEP	100%	**8.08** (5.39, 12.13)	**<2e‐16**	**7.55** (4.95, 11.52)	**<2e‐16**
PON3	99%	**0.69** (0.57, 0.83)	**0.000123**	**0.71** (0.58, 0.86)	**0.000450**
IGFBP1	98%	**0.74** (0.61, 0.90)	**0.002295**	**0.76** (0.62, 0.93)	**0.006683**
FABP4	96%	**1.91** (1.46, 2.51)	**2.95e‐06**	**2.02** (1.52, 2.68)	**9.68e‐07**
ADM	90%	**1.50** (1.16, 1.94)	**0.001884**	**1.55** (1.18, 2.03)	**0.001447**
GH	74%	**0.77** (0.63, 0.93)	**0.007026**	**0.81** (0.66, 1.00)	**0.044724**
PRSS27	69%	**0.73** (0.61, 0.86)	**0.000264**	**0.75** (0.63, 0.90)	**0.001481**
RAGE	63%	**0.70** (0.58, 0.85)	**0.000362**	**0.71** (0.58, 0.86)	**0.000668**

Combination low ASMM and high BFMI					
Model 1					
LEP	94%	**2.64** (1.84, 3.77)	**1.03e‐07**		
TIMP4	80%	**1.28** (1.03, 1.59)	**0.02760**		
CCL28	63%	**1.32** (1.08, 1.61)	**0.00712**		

ASMM, appendicular skeletal muscle mass; BFMI, body fat mass index; β, beta coefficient; CI, confidence interval; OR, odds ratio.

The cut point for variable selection in the boosting with stability selection was a selection frequency of 63%, which was determined by the algorithm based on the number of variables available for selection, the number of selected variables per iteration, and the maximum number of tolerable false positives.

Effect estimates have been calculated per 1 SD increase in normalized protein expression values on a log2 scale.

Model 1: adjustment for all 13 covariates (age, high‐density lipoprotein, triglycerides, glycated haemoglobin, estimated glomerular filtration rate, albumin, sex, physical activity, hypertension, smoking status, education, alcohol intake, and intake lipid‐lowering medication) as well as all other in the boosting with stability selection selected variables of the corresponding outcome.

Bold print indicates significance. Grey shading indicates change of the direction of association or attenuation of the association (i.e. non‐significant effect estimates) after adjustment in Model 2 compared with Model 1.

Concerning the association analysis, leptin (LEP) was the only protein biomarker that was selected for all five outcomes. Insulin‐like growth factor‐binding protein (IGFBP) 1 and 2, C‐C motif chemokine (CCL) 28, growth/differentiation factor 2 (GDF2), and growth hormone (GH) were selected for both, muscle and fat mass parameters. Kallikrein‐6 (KLK6), myoglobin (MB), and tissue factor pathway inhibitor (TFPI) were only selected for the two muscle mass parameters ASMM and low ASMM. Adrenomedullin (ADM), fatty acid‐binding protein 4 (FABP4), serine protease 27 (PRSS27), and paraoxonase (PON3) were only selected for the two fat mass parameters BFMI and high BFMI. LEP, CCL28, and metalloproteinase inhibitor 4 (TIMP4) were selected for the combination of low ASMM and high BFMI (*Table* 1).

*Table* 1 illustrates that after adjusting for the other outcome in Model 2, the associations of thrombospondin‐2 (THBS2) and GDF2 with ASMM, of CCL28 and IGFBP2 with BFMI as well as of GH with low ASMM became non‐significant. The association of LEP with ASMM was still significant but became inverse. After further including an interaction term between the above‐listed proteins and the other outcome, only the interaction between GDF2 and BFMI for the outcome of ASMM was significant [beta coefficient (β) (95% confidence interval, CI): −0.03 (−0.06, 0.00), *P* = 0.041].

### Cross‐sectional analysis: prediction of appendicular skeletal muscle mass/body fat mass index by classical risk factors and protein biomarkers

*Table* 2 displays the cross‐validated area under the curve (AUC) of a logistic regression model including 13 classical risk factors (AUC_basic_) and a model additionally including protein biomarkers (listed in Supporting information, *Table* S8) that were selected in ≥90% of the 100 group least absolute shrinkage and selection operator (lasso) bootstrap iterations (AUC_extended_) as well as their cross‐validated delta AUC (AUC_extended_ − AUC_basic_). The receiver operating characteristic (ROC) curves of the AUC cross‐validation are included in Supporting information, *Figure* S4.

**Table 2:** Prediction analysis — cross‐validated AUCs of logistic regression models with classical risk factors (mean AUC_basic_) and protein biomarkers in addition to classical risk factors (mean AUC_extended_)

Outcome	Mean AUC_basic_ (95% CI)	Mean AUC_extended_ (95% CI)	Mean delta AUC (95% CI)
Low ASMM	0.67 (0.65, 0.71)	0.83 (0.82, 0.87)	0.16 (0.13, 0.20)
High BFMI	0.67 (0.65, 0.72)	0.89 (0.88, 0.92)	0.22 (0.18, 0.25)
Combination low ASMM and high BFMI	0.73 (0.69, 0.80)	0.85 (0.83, 0.90)	0.12 (0.08, 0.17)

ASMM, appendicular skeletal muscle mass; AUC, area under the curve; BFMI, body fat mass index; CI, confidence interval.

AUC_basic_: AUC of a logistic regression model including 13 classical risk factors (age, high‐density lipoprotein, triglycerides, glycated haemoglobin, estimated glomerular filtration rate, albumin, sex, physical activity, hypertension, smoking status, education, alcohol intake, and intake lipid‐lowering medication). AUC_extended_: AUC of the basic model plus all protein biomarkers selected in ≥90% of the group least absolute shrinkage and selection operator bootstrap iterations (variables are listed in Supporting information, *Table* S8). Delta AUC: AUC_extended_ − AUC_basic_.

AUCs and delta AUCs are arithmetic means of 10‐fold cross‐validation. The confidence intervals of AUCs and delta AUCs were calculated via 100‐fold percentile bootstrapping.

**Figure 3:**
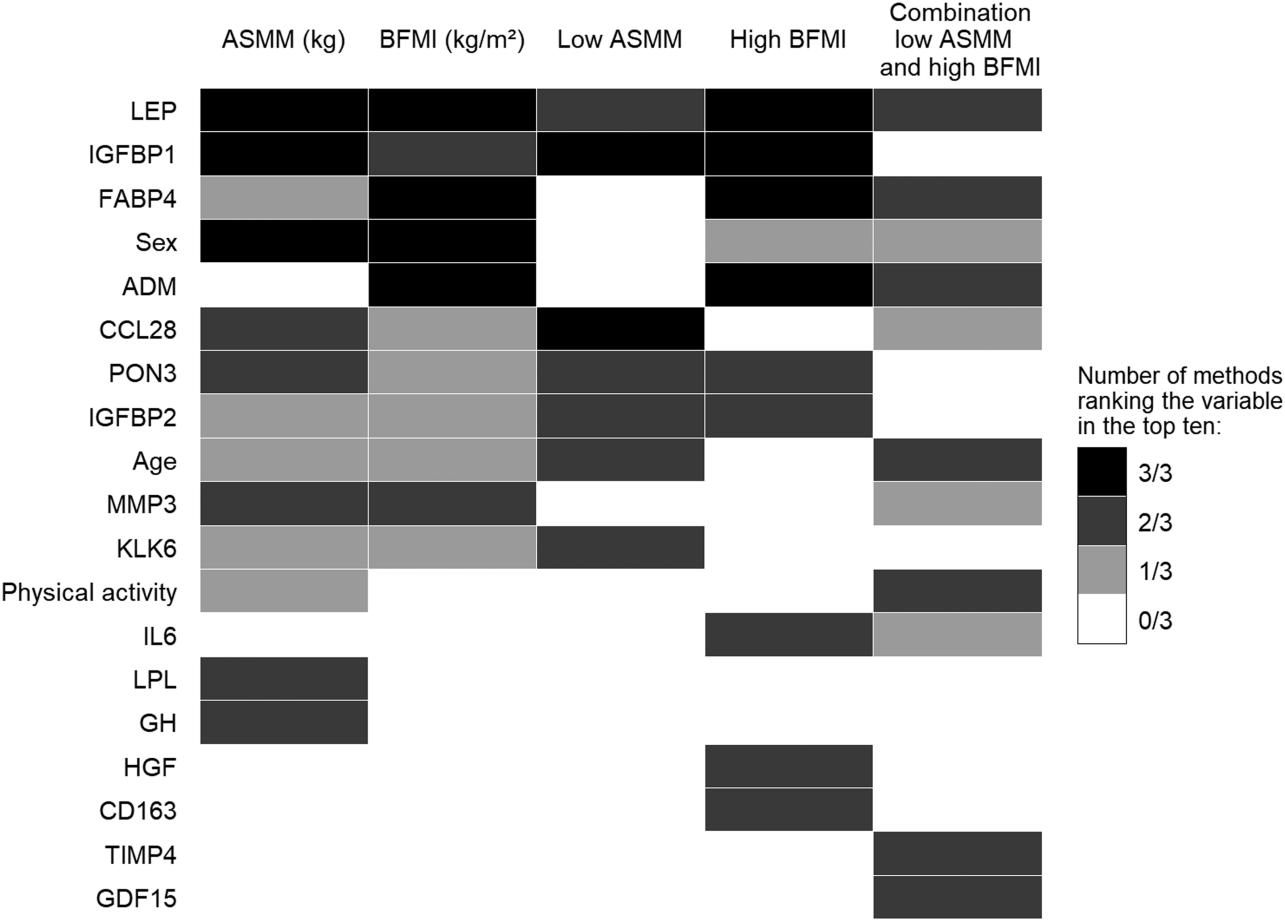
Sensitivity analysis — comparison of variables between the outcomes regarding the number of methods that ranked the variables in the top 10. Only variables are included that were ranked in the top 10 in at least two of the three analysis methods (group least absolute shrinkage and selection operator with 100× bootstrapping, random forest, and support vector machine) in at least one of the five outcomes. Variables are primarily ordered descending according to the total number (sum of all outcomes) of methods that ranked the variable in the top 10, and secondary according to the outcome in the table from left to right based on the number of methods that ranked the variable in the top 10 for the outcome. ASMM, appendicular skeletal muscle mass; BFMI, body fat mass index.

*Figure* 3 illustrates the results of the sensitivity analysis including the comparison of variables between the outcomes regarding the number of methods (group lasso with bootstrapping, random forest, and support vector machine) that ranked the variables in the top 10.

In the prediction analysis, the protein biomarkers were ranked equal to or even higher than classical risk factors (Supporting information, *Tables* S8 and S9) and were ranked in the top 10 in all three methods more consistently compared with classical risk factors (*Figure* 3).

### Longitudinal analysis

Detailed results regarding the longitudinal analysis are included in Supporting information, *Tables* S10–S13 and *Figure*s S5 and S6. Most relevant results of the association analysis include that N‐terminal prohormone brain natriuretic peptide (NT‐proBNP) was the only protein biomarker selected for a strong decrease in ASMM and the combination of a strong decrease in ASMM and a strong increase in BFMI. In logistic regression analyses, NT‐proBNP was positively associated with a strong decrease in ASMM [odds ratio (OR) (95% CI): 1.40 (1.10, 1.77) and the combined outcome (OR (95% CI): 1.60 (1.15, 2.24)] after adjustment for all 13 covariates. CCL4, CCL15, and a disintegrin and metalloproteinase with thrombospondin motifs 13 were selected for relative change in BFMI and protein delta homolog 1 for strong increase in BFMI. In the prediction analysis, group lasso with bootstrapping ranked NT‐proBNP in first place for both, strong decrease in ASMM and the combined outcome. Sensitivity analysis presents age for relative change in ASMM and CCL4 for relative change in BFMI as the only variables ranked in the top 10 of all three methods for any outcome.

## Discussion

This study aimed to identify new protein biomarkers of low muscle mass, high fat mass, and their combination as well as their changes over a 14 year follow‐up period. In our cross‐sectional analysis, we identified KLK6, CCL28, and TFPI as novel protein biomarkers associated with muscle mass and PRSS27 with fat mass. CCL28 and TIMP4 are newly detected biomarkers associated with the combination of low muscle and high fat mass. In the longitudinal analysis, NT‐proBNP was the only biomarker that was selected for a strong decrease in ASMM and the combination of a strong decrease in ASMM and a strong increase in BFMI over 14 years.

To the best of our knowledge, this is the first study to investigate the pathological condition of combined low muscle and high fat mass using proteomics. However, a few previous studies investigated related body composition parameters. Six studies investigated proteomics measured with PEA technology by Olink® using the CVDII panel with BMI‐defined obesity,^12^ inflammation panel with BMI and WC,^13^ CVDI panel with changes in BMI and WHR,^14^ inflammation, cardiometabolic, CVDII, and CVDIII panels with BMI‐defined obesity,^15^ immuno‐oncology panel with BMI,^19^ and a large‐scale mapping of genetics of the proteome investigated causal relationships of CVDI panel with BMI, body fat (%), and WHR.^18^ No previous study investigated PEA‐measured proteomics and muscle mass. Other studies implemented the aptamer‐based proteomics approach SOMAscan by SomaLogic (Boulder, Colorado, USA), which is as PEA a relative quantification method, but instead of antibodies, aptamers are used, which are randomly generated nucleotide sequences.^23^ Aptamer‐based proteomics were used to investigate dual‐energy X‐ray absorptiometry (DXA)‐measured body fat mass (kg)^16^ as well as DXA‐measured body fat (%) and lean body mass (kg).^17^

The comparison of our results with those of other studies has to be viewed with caution as different ethnicities can show varying body composition and numerous different parameters and measurement methods have been used to define muscle and fat mass. As BFMI showed strong correlations to BMI and WC in our data, comparisons of our results with those of studies using these parameters are feasible to some extent.

### Relevant protein biomarkers

In the cross‐sectional analysis, we identified various protein biomarkers associated with both, muscle and fat mass parameters, among them IGFBP1 and IGFBP2. In line with our results, both proteins are reduced with increasing obesity^24^ and an US‐American cohort study using single biomarkers measured with radioimmunoassays showed that higher total per cent fat and higher visceral fat were associated with lower IGFBP1 and IGFBP2.^25^ In a Swedish PEA‐based‐proteomics study, IGFBP1 was inversely associated with BMI‐defined obesity^15^ and by using aptamer methodology, IGFBP1 was inversely associated with fat mass (kg).^16^ Concerning muscle mass, IGFBP1 was inversely associated with DXA‐measured low relative muscle mass in a Swedish cohort of elderly women^26^ and IGFBP2 was inversely associated with DXA‐measured total muscle mass in an US‐American study.^27^ Both IGFBPs have been related to glucose and insulin levels and are known to be suppressed by GH.^24^ In our study, GH was selected for some of the same outcomes that the IGFBPs were selected for as well. A longitudinal PEA‐based study observed that a decrease in GH was associated with an increase in BMI and WHR over a 10 year period.^14^ Payette *et al*. summarized the characteristics of GH among others with a decreased secretion in obesity and in contrast to our results inducing anabolic effects on skeletal muscle. GH therapy can increase muscle mass, however with deleterious side effects.^28^ Even though the literature is clear regarding a positive association of GH and muscle mass, we observed an opposite association. This may be explained as follows: first, GH secretion is pulsatile and therefore difficult to interpret as an individual value measured at one time‐point.^29^ Second, under conditions of cachexia, that is, body wasting including a decrease in muscle and fat mass, observed usually in patients with chronic diseases such as heart failure (HF), GH resistance can develop.^30^ This is characterized by increased secretion of GH and reduced insulin‐like growth factor 1 (IGF‐1) as GH is ineffective in stimulating IGF‐1 production,^30^, ^31^ diminishing the highly relevant effect of IGF‐1 on muscle regeneration and decelerating muscle wasting under conditions of high GH concentrations.^30^ Even though we cannot prove this malfunction in our participants with low ASMM, this process might give an insight into the inverse relationship of GH with ASMM in our study.

KLK6, MB, and TFPI were only associated with the continuous and categorical parameters of ASMM. MB is an already known biomarker for increased muscle mass. MB further increases as a result of exercise induced through the degradation of protein structures within the muscle. In addition to its role in oxygen storage and transport, MB is thought to influence nitric oxide at the microvascular and tissue level.^32^ KLK6 and TFPI are new biomarkers associated with muscle mass. Due to a lack of previous studies related to body composition, we described the main hallmarks of the new biomarkers. The over‐expression of KLK6 transcript and protein has been recognized in numerous cancer types, such as breast, renal, pancreatic, ovarian, colorectal, and lung cancer.^33^ In our study, KLK6 was a risk factor for low muscle mass, notably, in participants without cancer. Moreover, KLK6 is linked to inflammatory pathways due to its ability to activate protease‐activated receptors, which are relevant in driving inflammatory processes. KLK6 is further attributed to participate in angiogenesis and apoptosis pathways.^33^ Regarding TFPI, recent articles investigated the biomarker as a potential treatment against haemophilia, due to its role in thrombin generation and coagulation processes.^34^ To our knowledge, the relations of KLK6 and TFPI to muscle mass have not been observed before.

FABP4, ADM, PRSS27, and PON3 were only associated with the continuous and categorical parameters of BFMI. In a previous PEA‐proteomics study, ADM and FABP4 were positively associated with BMI‐defined obesity.^15^ An increase in FABP4 was also associated with an increase in BMI and WHR over 10 years.^14^ Large‐scale mapping of genetics of the proteome identified that BMI and body fat (%) causally affected PEA‐measured ADM and FABP4 positively and WHR affected these biomarkers inversely.^18^ PRSS27 was the only new biomarker associated with fat mass in our study. The protease is largely unknown, and there are only a few articles mentioning PRSS27, for example, as a possible prognostic marker of oesophageal squamous cell carcinoma in patients with preoperative treatment.^35^

LEP, CCL28, and TIMP4 were associated with the combination of low ASMM and high BFMI. Associations of protein biomarkers to the combined outcome can only be expected if the associations to ASMM and BFMI are aligned in opposite directions or if the strengths of the associations differ to a high extent. If the associations of a biomarker to ASMM and BFMI are similar, a significant association to the combined outcome cannot be assumed as the combined outcome consists of opposite extremes (low ASMM and high BFMI). LEP is known to increase muscle mass and is inextricable from fat mass as it regulates energy expenditure and LEP sensitivity decreases in obesity.^36^ In relation to our results, previous European studies using PEA technology displayed that LEP was positively associated with BMI‐defined obesity^12^, ^15^ and changes in BMI and WHR.^14^ LEP measured with aptamer‐based proteomics was positively associated with fat mass (kg) in a European cohort^16^ and was selected as one of the top three proteins for body fat (%) but not lean mass (kg) in a Finnish cohort.^17^ In large‐scale mapping of genetics of the proteome, BMI and body fat (%) causally affected LEP positively and WHR affected LEP inversely.^18^ Underlying mechanisms connecting LEP to muscle and fat mass might constitute that under physiological conditions, LEP binds to its receptors in skeletal muscle and fat cells, which can initiate energy dissipation and reduce fatty acid accumulation as well as lipotoxicity in the muscle and fat cells. In obesity, LEP is increased but cannot bind to its receptors; thus, processes of fatty acid oxidation might be impaired, which can lead to intracellular accumulation of lipid intermediates.^36^ CCL28 is a new marker for muscle mass and as well as TIMP4 a new marker for the combination of low muscle and high fat mass. CCL28 has only recently and for the first time been inversely associated with the metabolic syndrome in Japanese adults also using PEA proteomics.^37^ Generally in line with this finding, we observed an inverse relationship of CCL28 with BFMI. TIMP4 is highly expressed in adipose tissue and was reported to promote high fat‐induced obesity, fatty liver, and dyslipidaemia in a study using TIMP4‐deficient mice exposed to high‐fat diet. The underlying mechanism may be the promotion of intestinal lipid absorption by TIMP4 through the reduction of the proteolytic processing of CD36, a fatty acid transporter in the small intestine. In addition, mice with deficient TIMP4 were protected against skeletal muscle triglyceride accumulation in the quadriceps.^38^ Our observations are in line with these reports, as higher levels of TIMP4 were associated with the combination of lower muscle mass and higher fat mass.

In the longitudinal analysis, NT‐proBNP was the only protein biomarker selected for a strong decrease in ASMM and the combination of a strong decrease in ASMM and a strong increase in BFMI. In a longitudinal cohort study, a decrease in NT‐proBNP was associated with an increase in BMI and WHR over 10 years.^14^ NT‐proBNP levels are increased in severe muscle wasting and the components of NT‐proBNP might be involved in lipolysis in adipose tissue.^39^ Furthermore, NT‐proBNP is already established in clinical application as a marker of HF. Muscle mass reduction as a part of body wasting is described as a complication of HF by the term cardiac cachexia.^30^ This could represent the linkage between higher baseline NT‐proBNP values and a stronger decrease in muscle mass over time. We are not able to directly verify this as PEA values are relative and not absolute protein concentrations necessary for HF classification. Additionally, compared with the association solely with a strong decrease in muscle mass, we observed a stronger association of NT‐proBNP with a strong decrease in muscle mass combined with a strong increase in fat mass, whereas cardiac cachexia is usually accompanied by reduced muscle and reduced fat mass. However, because not all HF patients show a decrease in fat mass as for instance over 80% of HF patients with preserved ejection fraction are overweight or obese,^30^ a decreased heart function reflected by NT‐proBNP could still be involved in this association.

### Cross‐sectional prediction analysis

Protein biomarkers were ranked equally high or even higher than most classical risk factors. In sensitivity analysis, protein biomarkers were ranked in the top 10 by all three methods more consistently compared with classical risk factors. The prediction performance reflected by the AUC for all three binary outcomes distinctly increased when protein biomarkers selected in ≥90% of group lasso bootstraps were added to the classical risk factors. This highlights the importance of protein biomarkers in addition to classical risk factors for optimal prediction of low muscle, high fat mass, and their combination.

### Comparison: cross‐sectional and longitudinal analysis

Prediction analysis in the longitudinal data yielded distinctly lower AUCs concerning all three, AUC_basic_ (only classical risk factors), AUC_extended_ (classical risk factors plus protein biomarkers), and delta AUC (AUC_extended_ − AUC_basic_), compared with the cross‐sectional data. Moreover, the overlap of biomarkers that were selected in both, longitudinal and cross‐sectional analyses, was lacking. The main reason likely is that our prospective data force the relation into the direction of baseline protein biomarkers leading to changes in body composition as proteomics data were only available at baseline, and we were therefore unable to investigate the changes in proteomics with the changes in body composition. If, in turn, body composition would affect the biomarkers, only cross‐sectional analyses without pre‐specified direction would be able to identify the association. A recently published large‐scale mapping of genetics of the proteome supports this concept as it demonstrated that body fat (%) causally affected LEP, ADM, and FABP4, but there was only weak evidence of FABP4 and LEP and no evidence of ADM causally affecting body fat (%).^18^ In our analysis, these biomarkers were selected in cross‐sectional but not in longitudinal analysis, possibly due to the lacking causal effect of the biomarkers on fat mass.

### Strengths and limitations

A major strength of this project presents the usage of proteomics in addition to classical risk factors enabling us to simultaneously analyse 233 protein biomarkers. Furthermore, we employed multiple machine learning approaches to analyse the data based on different aspects. Another strength encompasses the usage of stability selection, which strongly minimizes false positives in the association analysis. Only protein biomarkers with good measurement quality were included in the analysis. Concerning the comparison of the outcomes, bias was minimized as muscle and fat mass were calculated based on the same BIA measurements. Another strength constitutes the implementation of both cross‐sectional and longitudinal approaches.

A few limitations of the present study also require acknowledgement. First, generalizability of the results is limited for younger adults and other ethnicities, because the study included primarily white Europeans aged 55–74 years. Second, as the number of participants in the combined outcome was relatively low, we had to implement different cut points for the combined outcome compared with the single outcomes. Third, the PEA technique used for proteomics measurements provides only relative and not absolute protein concentration. Fourth, as we used a targeted proteomics approach with proteins selected for inflammation and CVDs, other non‐targeted proteins could also be relevant for muscle and fat mass.

## Conclusion

To the best of our knowledge, we identified KLK6, CCL28, and TFPI as novel protein biomarkers associated with muscle mass and PRSS27 with fat mass. CCL28 and TIMP4 are new biomarkers associated with the combination of a low muscle and a high fat mass. NT‐proBNP was the only biomarker selected for a strong decrease in muscle mass and the combination of a strong decrease in muscle mass and a strong increase in fat mass over 14 years. In the cross‐sectional analysis, proteomics substantially improved the prediction of low muscle, high fat mass, and their combination on top of classical risk factors.

## Funding

The KORA study was initiated and financed by the Helmholtz Zentrum München– German Research Center for Environmental Health, which is funded by the German Federal Ministry of Education and Research (BMBF) and by the State of Bavaria. The present study was supported by Helmholtz Institute for Metabolic, Obesity and Vascular Research – Project Initiative 2018 (HI‐MAG).

## Conflict of interest

Marie‐Theres Huemer, Alina Bauer, Agnese Petrera, Markus Scholz, Stefanie M. Hauck, Michael Drey, Annette Peters, and Barbara Thorand declare that they have no conflict of interest.

## Ethical guidelines statement

The authors of this manuscript certify that they comply with the ethical guidelines for authorship and publishing in the *Journal of Cachexia, Sarcopenia and Muscle*.^40^ All study methods were approved by the ethics committee of the Bavarian Chamber of Physicians, Munich (S4: EC No. 99186, FF4: EC No. 06068), and were performed in accordance with the ethical standards laid down in the 1964 Declaration of Helsinki and its later amendments. All participants gave their written informed consent.

## Supporting information


**Figure S1.** Flow chart of participant exclusions of cross‐sectional and longitudinal analysis
Details regarding exclusions of the protein biomarkers
**Figure S2**. Biomarker exclusions in the three proteomics panels
**Table S1**. Biomarker information CVDII panel
**Table S2**. Biomarker information CVDIII panel
**Table S3**. Biomarker information Inflammation panel
Detailed description concerning the calculations of the outcomes
**Table S4**. Definition of the outcomes in the cross‐sectional analysis
**Figure S3**. Definition of the outcomes in the cross‐sectional analysis
**Table S5**. Definition of the outcomes in the longitudinal analysis
Detailed description of the covariates
Detailed description of the statistical analysis
**Table S6**. Baseline (S4) characteristics of the study population
**Table S7.** Characteristics of the study population in the longitudinal sample
**Table S8.** Cross‐sectional analysis – Prediction analysis – Group lasso with 100x bootstrapping
**Figure S4.** Smoothed ROC curves of 10‐fold cross‐validation of logistic regression models with classical risk factors (AUC_basic_) and protein biomarkers in addition to classical risk factors (AUC_extended_)
**Table S9.** Cross‐sectional analysis – Sensitivity analysis – Comparison of the top 10 most important variables of lasso, random forest, and support vector machine
Results of the longitudinal analysis
**Table S10.** Association analysis – Boosting with stability selection – Longitudinal analysis
**Figure S5**. Association analysis – Boosting with stability selection – Comparison of protein biomarker selection between the outcomes – Longitudinal analysis
**Table S11.** Prediction analysis – Group lasso with 100x bootstrapping – Longitudinal analysis
**Table S12.** Prediction analysis – Cross‐validated AUCs of logistic regression models with classical risk factors (mean AUC_basic_) and protein biomarkers in addition to classical risk factors (mean AUC_extended_) – Longitudinal analysis
**Table S13.** Sensitivity Analysis – Comparison of the top 10 most important variables of lasso, random forest, and support vector machine – Longitudinal analysis
**Figure S6.** Sensitivity Analysis – Comparison of variables between the outcomes regarding the number of methods that ranked the variables in the top ten – Longitudinal analysis
