# Supplementary material for: Proteomic profiling of low muscle and high fat mass: a machine learning approach in the KORA S4/FF4 study
Source: J Cachexia Sarcopenia Muscle. 2021 Jun 20;12(4):1011–23. doi: 10.1002/jcsm.12733 (PMC8350207; doi:10.1002/jcsm.12733)
Supplement: Supplementary file 1 — Figure S1. Flow chart of participant exclusions of cross‐sectional and longitudinal analysis Details regarding exclusions of the protein biomarkers Figure S2. Biomarker exclusions in the three proteomics panels Table S1. Biomarker information CVDII panel Table S2. Biomarker information CVDIII panel Table S3. Biomarker information Inflammation panel Detailed description concerning the calculations of the outcomes Table S4. Definition of the outcomes in the cross‐sectional analysis Figure S3. Definition of the outcomes in the cross‐sectional analysis Table S5. Definition of the outcomes in the longitudinal analysis Detailed description of the covariates Detailed description of the statistical analysis Table S6. Baseline (S4) characteristics of the study population Table S7. Characteristics of the study population in the longitudinal sample Table S8. Cross‐sectional analysis – Prediction analysis – Group lasso with 100x bootstrapping Figure S4. Smoothed ROC curves of 10‐fold cross‐validation of logistic regression models with classical risk factors (AUCbasic) and protein biomarkers in addition to classical risk factors (AUCextended) Table S9. Cross‐sectional analysis – Sensitivity analysis – Comparison of the top 10 most important variables of lasso, random forest, and support vector machine Results of the longitudinal analysis Table S10. Association analysis – Boosting with stability selection – Longitudinal analysis Figure S5. Association analysis – Boosting with stability selection – Comparison of protein biomarker selection between the outcomes – Longitudinal analysis Table S11. Prediction analysis – Group lasso with 100x bootstrapping – Longitudinal analysis Table S12. Prediction analysis – Cross‐validated AUCs of logistic regression models with classical risk factors (mean AUCbasic) and protein biomarkers in addition to classical risk factors (mean AUCextended) – Longitudinal analysis Table S13. Sensitivity Analysis – Comparison of the top 10 most important varia [file JCSM-12-1011-s001.pdf]

Supporting information

-

Proteomic profiling of low muscle and high fat mass: a machine learning approach in the KORA S4/FF4 study

Marie-Theres Huemer[1], Alina Bauer[1], Agnese Petrera[2], Markus Scholz[3], Stefanie M. Hauck[2], Michael Drey[4], Annette Peters[1,5,6], Barbara Thorand[1,5]

[1]Institute of Epidemiology, Helmholtz Zentrum München, German Research Center for Environmental Health (GmbH), Neuherberg, Germany

[2]Research Unit Protein Science, Helmholtz Zentrum München, German Research Center for Environmental Health (GmbH), Neuherberg, Germany

[3]Institute for Medical Informatics, Statistics and Epidemiology (IMISE), Universität Leipzig, Leipzig, Germany

[4]Medizinische Klinik und Poliklinik IV, Schwerpunkt Akutgeriatrie, Klinikum der Universität München (LMU), Munich, Germany

[5]German Center for Diabetes Research (DZD), München-Neuherberg, Germany

[6]Chair of Epidemiology, Institute for Medical Information Processing, Biometry and Epidemiology, Medical Faculty, Ludwig-Maximilians-Universität München, Munich, Germany

Corresponding author:

Prof. Dr. Barbara Thorand

Email: thorand@helmholtz-muenchen.de

Phone: ++49-(0)89-3187-4480

Institute of Epidemiology

Helmholtz Zentrum München, German Research Center for Environmental Health (GmbH)

Ingolstaedter Landstraße 1

D-85764 Neuherberg, Germany

Supporting information

Figure S1: Flow chart of participant exclusions of cross-sectional and longitudinal analysis

Details regarding exclusions of the protein biomarkers

Figure S2: Biomarker exclusions in the three proteomics panels

Table S1: Biomarker information CVDII panel

Table S2: Biomarker information CVDIII panel

Table S3: Biomarker information Inflammation panel

Detailed description concerning the calculations of the outcomes

Table S4: Definition of the outcomes in the cross-sectional analysis

Figure S3: Definition of the outcomes in the cross-sectional analysis

Table S5: Definition of the outcomes in the longitudinal analysis

Detailed description of the covariates

Detailed description of the statistical analysis

Table S6: Baseline (S4) characteristics of the study population

Table S7: Characteristics of the study population in the longitudinal sample

Table S8: Cross-sectional analysis – Prediction analysis – Group lasso with 100x bootstrapping

Figure S4: Smoothed ROC curves of 10-fold cross-validation of logistic regression models with classical risk factors (AUC_{basic}) and protein biomarkers in addition to classical risk factors ($AUC_{extended}$)

Table S9: Cross-sectional analysis – Sensitivity analysis – Comparison of the top 10 most important variables of lasso, random forest, and support vector machine

Results of the longitudinal analysis

Table S10: Association analysis – Boosting with stability selection – Longitudinal analysis

Figure S5: Association analysis – Boosting with stability selection – Comparison of protein biomarker selection between the outcomes – Longitudinal analysis

Table S11: Prediction analysis – Group lasso with 100x bootstrapping – Longitudinal analysis

Table S12: Prediction analysis – Cross-validated AUCs of logistic regression models with classical risk factors (mean AUC_{basic}) and protein biomarkers in addition to classical risk factors (mean $AUC_{extended}$) – Longitudinal analysis

Table S13: Sensitivity Analysis – Comparison of the top 10 most important variables of lasso, random forest, and support vector machine – Longitudinal analysis

Figure S6: Sensitivity Analysis – Comparison of variables between the outcomes regarding the number of methods that ranked the variables in the top 10 – Longitudinal analysis

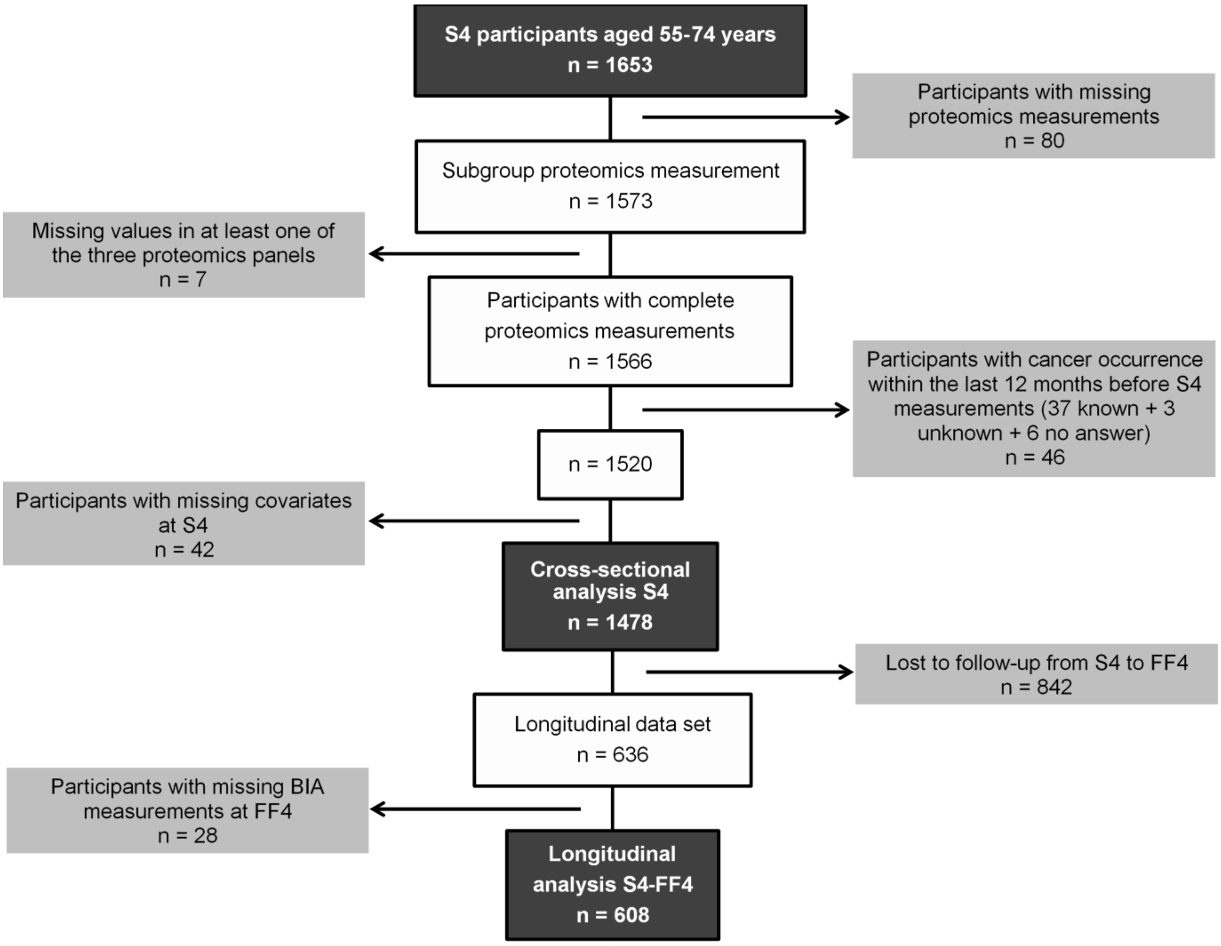

Figure S1: Flow chart of participant exclusions of cross-sectional and longitudinal analysis

BIA, bioelectrical impedance analysis; n, number of participants.

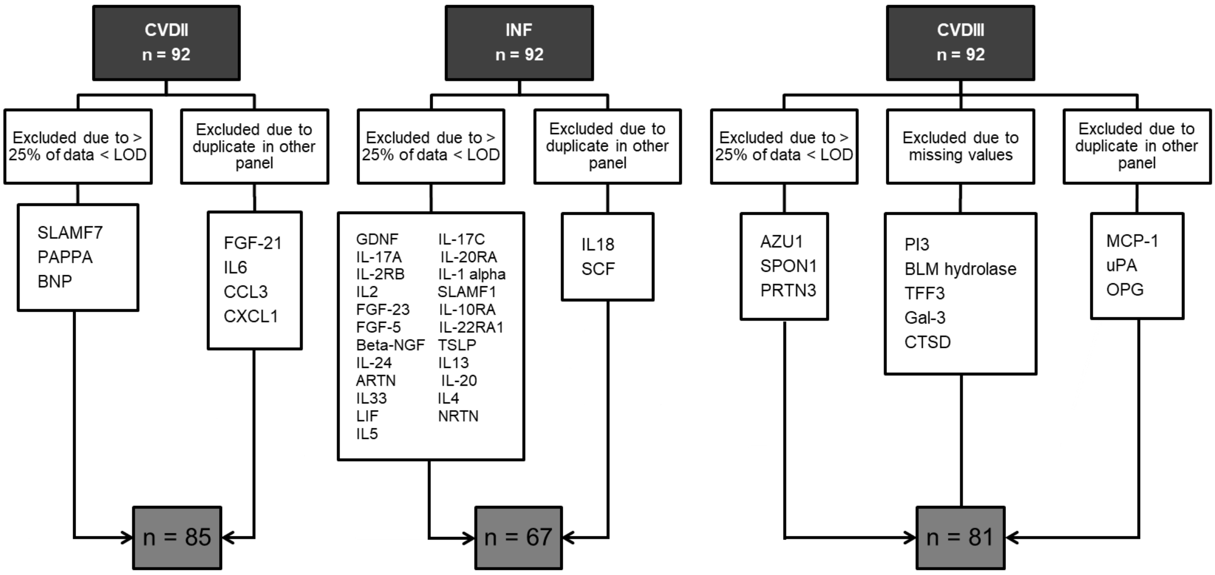

Figure S2: Biomarker exclusions in the three proteomics panels

CVD, cardiovascular disease; INF, inflammation; LOD, limit of detection; n, number of protein biomarkers.

Details regarding exclusions of the protein biomarkers:

We excluded three biomarkers of panel CVDII, three biomarkers of the panel CVDIII, and 23 biomarkers of the panel Inflammation due to values below the limit of detection (LOD) in > 25 % of all data before participant exclusions. From the remaining data, nine biomarkers were measured in duplicate for all participants in two different panels: Six biomarkers were enclosed in both CVDII and Inflammation panels and three biomarkers were included in both CVDIII and Inflammation panels. We decided to exclude the values of the panel in which the data entailed more values below the LOD and if not applicable, a higher inter-assay coefficient of variation. Resulting from this, four biomarkers of CVDII, three biomarkers of CVDIII, and two biomarkers of Inflammation were excluded. Additionally, five biomarkers were excluded in CVDIII, because of missing values not resulting from values below LOD. This concludes to a total number of 233 different protein biomarkers incorporated into the analysis (Figure S2). For all biomarkers that were not excluded and contained values < LOD, the values < LOD remained in the data and were not substituted.

Table S1: Biomarker information CVDII panel

Biomarker ID	Full name	UniProt ID	LOD	Values <LOD (n)	Values <LOD (%)	Intra-Assay CV (%)	Inter-Assay CV (%)
ACE2	Angiotensin-converting enzyme 2	Q9BYF1	1.18	0	0	11	13.73
ADAM-TS13	A disintegrin and metalloproteinase with thrombospondin motifs 13	Q76LX8	1.88	0	0	5.58	6.92
ADM	Adrenomedullin	P35318	1.06	0	0	10.15	12.5
AGRP	Agouti-related protein	O00253	0.47	0	0	5.16	12.19
AMBP	Protein AMBP	P02760	0.83	0	0	3.42	4.93
ANGPT1	Angiopoietin-1	Q15389	0.86	0	0	6.82	23.38
BMP-6	Bone morphogenetic protein 6	P22004	2.08	0	0	5.98	17.1
BNP	Natriuretic peptides B	P16860	1.55	1092	69.55	[a]	[a]
BOC	Brother of CDO	Q9BWV1	0.61	0	0	6.77	10.52
CA5A	Carbonic anhydrase 5A, mitochondrial	P35218	1.51	317	20.19	11.19	14.66

Biomarker ID	Full name	UniProt ID	LOD	Values <LOD (n)	Values <LOD (%)	Intra-Assay CV (%)	Inter-Assay CV (%)
CCL17	C-C motif chemokine 17	Q92583	0.83	0	0	6.11	30.36
CCL3	C-C motif chemokine 3	P10147	0.58	0	0	5.58	13.28
CD4	T-cell surface glycoprotein CD4	P01730	1.26	0	0	5.65	15.22
CD40-L	CD40 ligand	P29965	2.46	4	0.25	6.73	63.53
CD84	SLAM family member 5	Q9UIB8	2.08	0	0	4.64	18.96
CEACAM8	Carcinoembryonic antigenrelated cell adhesion molecule 8	P31997	1.91	0	0	5.55	16.21
CTRC	Chymotrypsin C	Q99895	1.42	0	0	5.15	10.92
CTSL1	Cathepsin L1	P07711	0.5	0	0	4.06	10.3
CXCL1	C-X-C motif chemokine 1	P09341	3.84	0	0	4.09	43.33
DCN	Decorin	P07585	1.13	0	0	3.86	9.15
DECR1	2,4-dienoyl-CoA reductase, mitochondrial	Q16698	4.95	25	1.59	12.07	39.82
Dkk-1	Dickkopf-related protein 1	O94907	0.93	0	0	5.11	16.65
FABP2	Fatty acid-binding protein, intestinal	P12104	1.66	0	0	5.38	15.76
FGF-21	Fibroblast growth factor 21	Q9NSA1	1.84	0	0	7.12	13.39
FGF-23	Fibroblast growth factor 23	Q9GZV9	2.96	28	1.78	4.22	10.24
FS	Follistatin	P19883	2.08	0	0	4.26	9.51
Gal-9	Galectin-9	O00182	0.46	0	0	3.57	8.49
GDF-2	Growth/differentiation factor 2	Q9UK05	2.81	0	0	7.02	12.5
GH	Growth hormone	P01241	1.1	0	0	3.7	28.29
GIF	Gastric intrinsic factor	P27352	1.7	3	0.19	10.09	11.65
GLO1	Lactoylglutathione lyase	Q04760	1.86	0	0	5.67	66.17
GT	Gastrotropin	P51161	0.75	34	2.17	6.18	11.21
HAOX1	Hydroxyacid oxidase 1	Q9UJM8	1.1	0	0	8.89	17.66
HB-EGF	Proheparin-binding EGF-like growth factor	Q99075	0.61	0	0	6.36	17.6
HO-1	Heme oxygenase 1	P09601	0.97	0	0	5.84	9.94
hOSCAR	Osteoclast-associated immunoglobulin-like receptor	Q8IYS5	2.29	0	0	6.19	6.87
HSP 27	Heat shock 27 kDa protein	P04792	4.29	0	0	5.26	8.73
IDUA	Alpha-L-iduronidase	P35475	1.73	0	0	5.5	15.53

Biomarker ID	Full name	UniProt ID	LOD	Values <LOD (n)	Values <LOD (%)	Intra-Assay CV (%)	Inter-Assay CV (%)
IgG Fc receptor II-b	Low affinity immunoglobulin gamma Fc region receptor II-b	P31994	2.2	122	7.77	7.24	13.47
IL-17D	Interleukin-17D	Q8TAD2	1.95	52	3.31	9.15	12.73
IL-1RA	Interleukin-1 receptor antagonist protein	P18510	1.79	0	0	4.49	18.48
IL-27	Interleukin-27	Q8NEV9,Q14213	0.99	0	0	4.56	6.7
IL-4RA	Interleukin-4 receptor subunit alpha	P24394	0.88	0	0	4.91	16.73
IL16	Pro-interleukin-16	Q14005	2.47	0	0	7.43	71.9
IL18	Interleukin-18	Q14116	0.45	0	0	5.68	10.41
IL1RL2	Interleukin-1 receptor-like 2	Q9HB29	2.14	0	0	7.33	12.14
IL6	Interleukin-6	P05231	1.57	50	3.18	3.98	11.3
ITGB1BP2	Melusin	Q9UKP3	4.89	123	7.83	[a]	9.72
KIM1	Kidney Injury Molecule	Q96D42	2.01	0	0	5.14	10.28
LEP	Leptin	P41159	1.59	0	0	5.82	14.32
LOX-1	Lectin-like oxidized LDL receptor 1	P78380	1.08	0	0	4.87	12.58
LPL	Lipoprotein lipase	P06858	2.36	0	0	3.13	7.99
MARCO	Macrophage receptor MARCO	Q9UEW3	1.42	0	0	4.16	11.34
MERTK	Tyrosine-protein kinase Mer	Q12866	2.08	0	0	4.84	12.96
MMP12	Matrix metalloproteinase-12	P39900	0.76	0	0	4.62	10.53
MMP7	Matrix metalloproteinase-7	P09237	1.36	0	0	3.31	22.69
NEMO	NF-kappa-B essential modulator	Q9Y6K9	3.74	2	0.13	7.78	68.32
PAPPA	Pappalysin-1	Q13219	2.99	1229	78.28	8.53	8.92
PAR-1	Proteinase-activated receptor 1	P25116	1.38	0	0	5.33	23.68
PARP-1	Poly [ADP-ribose] polymerase 1	P09874	3.96	315	20.06	24.09	19.74
PD-L2	Programmed cell death 1 ligand 2	Q9BQ51	1.55	2	0.13	5.13	10.54
PDGF subunit B	Platelet-derived growth factor subunit B	P01127	2.26	0	0	7.82	25.22
PGF	Placenta growth factor	P49763	1.08	0	0	8.32	11.87
PIgR	Polymeric immunoglobulin receptor	P01833	2.15	0	0	4.19	6.07
PRELP	Prolargin	P51888	0.39	0	0	3.89	6.32
PRSS27	Serine protease 27	Q9BQR3	0.62	0	0	4.02	6.18

Biomarker ID	Full name	UniProt ID	LOD	Values <LOD (n)	Values <LOD (%)	Intra-Assay CV (%)	Inter-Assay CV (%)
PRSS8	Prostasin	Q16651	2.1	0	0	3.75	7.54
PSGL-1	P-selectin glycoprotein ligand 1	Q14242	1.12	0	0	6.16	7.38
PTX3	Pentraxin-related protein PTX3	P26022	1.6	0	0	6.27	9.3
RAGE	Receptor for advanced glycosylation end products	Q15109	1.15	0	0	4.97	10.14
REN	Renin	P00797	0.76	0	0	7.56	10.6
SCF	Stem cell factor	P21583	0.89	0	0	3.26	7
SERPINA12	Serpin A12	Q8IW75	-0.16	2	0.13	5.18	13.01
SLAMF7	SLAM family member 7	Q9NQ25	3.51	498	31.72	6.97	13.61
SOD2	Superoxide dismutase [Mn], mitochondrial	P04179	0.66	0	0	3.86	4.91
SORT1	Sortilin	Q99523	1.45	0	0	3.47	8.43
SPON2	Spondin-2	Q9BUD6	0.62	0	0	4.14	7.96
SRC	Proto-oncogene tyrosine-protein kinase Src	P12931	1.29	0	0	5.37	29.18
STK4	Serine/threonine-protein kinase 4	Q13043	2.64	15	0.96	7.99	26.11
TF	Tissue factor	P13726	0.06	0	0	4.2	8.53
TGM2	Protein-glutamine gamma-glutamyltransferase 2	P21980	2.44	0	0	2.43	12.44
THBS2	Thrombospondin-2	P35442	0.29	0	0	3.67	4.81
THPO	Thrombopoietin	P40225	0.14	0	0	5.72	11.97
TIE2	Angiopoietin-1 receptor	Q02763	1.43	0	0	5.24	7.67
TM	Thrombomodulin	P07204	3.73	0	0	4.74	8.32
TNFRSF10A	Tumor necrosis factor receptor superfamily member 10A	O00220	2.01	0	0	5.44	9.71
TNFRSF11A	Tumor necrosis factor receptor superfamily member 11A	Q9Y6Q6	1.24	0	0	4.9	10.96
TNFRSF13B	Tumor necrosis factor receptor superfamily member 13B	O14836	1.84	0	0	4.75	8.33
TRAIL-R2	TNF-related apoptosis-inducing ligand receptor 2	O14763	1.68	0	0	6.33	9.24
VEGFD	Vascular endothelial growth factor D	O43915	0.32	0	0	5.61	6.81
VSIG2	V-set and immunoglobulin domain-containing protein 2	Q96IQ7	2.16	0	0	7.83	11.65

Biomarker ID	Full name	UniProt ID	LOD	Values <LOD (n)	Values <LOD (%)	Intra-Assay CV (%)	Inter-Assay CV (%)
XCL1	Lymphotactin	P47992	0.5	0	0	4.61	10.06

CV, coefficient of variation; LOD, limit of detection; UniProt ID, universal protein database identification.

[a] All or nearly all values of the control samples, which are requisite to calculate the CVs, were < LOD. If the values of the control samples are < LOD, they are not included in the calculation of the CVs. Therefore, the number of available values was too low to estimate the CV.

Table S2: Biomarker information CVDIII panel

Biomarker ID	Full name	UniProt ID	LOD	Values <LOD (n)	Values <LOD (%)	Intra-Assay CV (%)	Inter-Assay CV (%)
ALCAM	CD166 antigen	Q13740	0.71	0	0	6.04	16.21
AP-N	Aminopeptidase N	P15144	0.63	0	0	5.42	13.16
AXL	Tyrosine-protein kinase receptor UFO	P30530	2.95	0	0	6.62	18.45
AZU1	Azurocidin	P20160	3.06	841	53.46	[a]	[a]
BLM hydrolase	Bleomycin hydrolase	Q13867	1.45	52	3.31	5.41	18.12
CASP-3	Caspase-3	P42574	3.14	4	0.25	6.18	69.48
CCL15	C-C motif chemokine 15	Q16663	0.76	0	0	6.81	17.15
CCL16	C-C motif chemokine 16	O15467	0.58	0	0	8.22	19.65
CCL24	C-C motif chemokine 24	O00175	0.61	0	0	6.45	17.3
CD163	Scavenger receptor cysteine-rich type 1 protein M130	Q86VB7	0.88	0	0	6.62	16.62
CD93	Complement component C1q receptor	Q9NPY3	1.21	0	0	5.14	15.99
CDH5	Cadherin-5	P33151	1.24	0	0	7.06	18.09
CHI3L1	Chitinase-3-like protein 1	P36222	2.86	164	10.43	4.95	13.02
CHIT1	Chitotriosidase-1	Q13231	1.58	87	5.53	5.22	17.57
CNTN1	Contactin-1	Q12860	0.69	0	0	7.06	17.89
COL1A1	Collagen alpha-1(I) chain	P02452	0.35	0	0	5.16	14.62
CPA1	Carboxypeptidase A1	P15085	0.40	0	0	5.61	13.75
CPB1	Carboxypeptidase B	P15086	0.20	0	0	5.64	13.64
CSTB	Cystatin-B	P04080	3.26	47	2.99	6.83	22.64
CTSD	Cathepsin D	P07339	1.26	0	0	4.51	14.86

Biomarker ID	Full name	UniProt ID	LOD	Values <LOD (n)	Values <LOD (%)	Intra-Assay CV (%)	Inter-Assay CV (%)
CTSZ	Cathepsin Z	Q9UBR2	0.64	0	0	5.51	15.3
CXCL16	C-X-C motif chemokine 16	Q9H2A7	0.69	0	0	5.42	15.85
DLK-1	Protein delta homolog 1	P80370	0.62	0	0	6.24	17.52
EGFR	Epidermal growth factor receptor	P00533	0.90	0	0	5.66	13.34
Ep-CAM	Epithelial cell adhesion molecule	P16422	2.29	0	0	5.69	21.07
EPHB4	Ephrin type-B receptor 4	P54760	1.40	0	0	5.17	15.22
FABP4	Fatty acid-binding protein 4	P15090	1.61	0	0	5.85	21.23
FAS	Tumor necrosis factor receptor superfamily member 6	P25445	-0.16	0	0	5.81	16.09
Gal-3	Galectin-3	P17931	0.61	0	0	5.64	14.55
Gal-4	Galectin-4	P56470	0.61	0	0	7.03	14.98
GDF-15	Growth/differentiation factor 15	Q99988	0.51	0	0	5.07	14.89
GP6	Platelet glycoprotein VI	Q9HCN6	1.00	37	2.35	5.82	34.23
GRN	Granulins	P28799	1.97	0	0	5.44	13.83
ICAM-2	Intercellular adhesion molecule 2	P13598	1.10	0	0	6.59	15.94
IGFBP-1	Insulin-like growth factor-binding protein 1	P08833	1.33	0	0	5.44	17.38
IGFBP-2	Insulin-like growth factor-binding protein 2	P18065	1.54	0	0	5.52	17.79
IGFBP-7	Insulin-like growth factor-binding protein 7	Q16270	1.19	0	0	6.37	16.87
IL-17RA	Interleukin-17 receptor A	Q96F46	1.36	0	0	6.3	24.57
IL-18BP	Interleukin-18-binding protein	O95998	0.90	0	0	6.13	15.4
IL-1RT1	Interleukin-1 receptor type 1	P14778	2.17	0	0	5.74	15.31
IL-1RT2	Interleukin-1 receptor type 2	P27930	2.85	0	0	5.66	14.4
IL-6RA	Interleukin-6 receptor subunit alpha	P08887	2.39	0	0	5.22	15.24
IL2-RA	Interleukin-2 receptor subunit alpha	P01589	0.56	0	0	5.13	13.17
ITGB2	Integrin beta-2	P05107	3.61	0	0	5.04	31.13
JAM-A	Junctional adhesion molecule A	Q9Y624	2.45	1	0.06	7.28	64.49
KLK6	Kallikrein-6	Q92876	1.59	75	4.77	6.87	14.04
LDL receptor	Low-density lipoprotein receptor	P01130	0.73	0	0	5.71	16.18
LTBR	Lymphotoxin-beta receptor	P36941	0.42	0	0	4.95	15.07

Biomarker ID	Full name	UniProt ID	LOD	Values <LOD (n)	Values <LOD (%)	Intra-Assay CV (%)	Inter-Assay CV (%)
MB	Myoglobin	P02144	2.16	0	0	6.48	15.06
MCP-1	Monocyte chemotactic protein 1	P13500	0.49	0	0	5.65	19.24
MEPE	Matrix extracellular phosphoglycoprotein	Q9NQ76	1.11	0	0	8.96	19.91
MMP-2	Matrix metalloproteinase-2	P08253	0.40	0	0	6.31	18.11
MMP-3	Matrix metalloproteinase-3	P08254	1.30	0	0	6.64	17.82
MMP-9	Matrix metalloproteinase-9	P14780	2.06	0	0	6.13	23.75
MPO	Myeloperoxidase	P05164	3.22	347	22.06	3.73	12.41
Notch 3	Neurogenic locus notch homolog protein 3	Q9UM47	0.87	0	0	7.24	18.32
NT-proBNP	N-terminal prohormone brain natriuretic peptide	NA	2.34	236	15	6.62	17.19
OPG	Osteoprotegerin	O00300	0.73	0	0	5.78	15.64
OPN	Osteopontin	P10451	1.04	0	0	6.22	19.29
PAI	Plasminogen activator inhibitor 1	P05121	1.35	0	0	5.89	22.87
PCSK9	Proprotein convertase subtilisin/kexin type 9	Q8NBP7	0.82	0	0	8.13	18.11
PDGF subunit A	Platelet-derived growth factor subunit A	P04085	2.08	57	3.62	5.5	36.9
PECAM-1	Platelet endothelial cell adhesion molecule	P16284	0.99	0	0	5.32	37.48
PGLYRP1	Peptidoglycan recognition protein 1	O75594	1.63	0	0	5.36	16.09
PI3	Elafin	P19957	1.13	66	4.2	10.15	38.14
PLC	Perlecan	P98160	3.40	0	0	4.73	14.56
PON3	Paraoxonase	Q15166	0.68	0	0	6.92	18.11
PRTN3	Myeloblastin	P24158	3.66	429	27.27	4.07	21.1
PSP-D	Pulmonary surfactant-associated protein D	P35247	1.40	16	1.02	8.65	12.08
RARRES2	Retinoic acid receptor responder protein 2	Q99969	1.39	0	0	7.64	14.89
RETN	Resistin	Q9HD89	2.85	0	0	5.26	15.52
SCGB3A2	Secretoglobin family 3A member 2	Q96PL1	0.20	0	0	6.24	17.44
SELE	E-selectin	P16581	3.23	0	0	4.84	13.58
SELP	P-selectin	P16109	1.92	0	0	6.47	40.36
SHPS-1	Tyrosine-protein phosphatase non-receptor type substrate 1	P78324	1.04	0	0	6.23	17.31
SPON1	Spondin-1	Q9HCB6	1.51	746	47.43	5.6	10.9

Biomarker ID	Full name	UniProt ID	LOD	Values <LOD (n)	Values <LOD (%)	Intra-Assay CV (%)	Inter-Assay CV (%)
ST2	ST2 protein	Q01638	1.62	1	0.06	8.81	14.62
t-PA	Tissue-type plasminogen activator	P00750	2.44	0	0	5.6	25.63
TFF3	Trefoil factor 3	Q07654	2.68	0	0	6.49	15.5
TFPI	Tissue factor pathway inhibitor	P10646	0.53	0	0	6.2	15.91
TIMP4	Metalloproteinase inhibitor 4	Q99727	0.58	0	0	5.78	14.53
TLT-2	Trem-like transcript 2 protein	Q5T2D2	2.47	0	0	7.23	22
TNF-R1	Tumor necrosis factor receptor 1	P19438	1.36	0	0	6.46	15.15
TNF-R2	Tumor necrosis factor receptor 2	P20333	2.17	0	0	6.08	15.06
TNFRSF10C	Tumor necrosis factor receptor superfamily member 10C	O14798	1.77	0	0	5.94	14.54
TNFRSF14	Tumor necrosis factor receptor superfamily member 14	Q92956	1.85	0	0	5.39	18.65
TNFSF13B	Tumor necrosis factor ligand superfamily member 13B	Q9Y275	1.28	0	0	6.02	16.91
TR	Transferrin receptor protein 1	P02786	0.57	0	0	4.34	12.57
TR-AP	Tartrate-resistant acid phosphatase type 5	P13686	1.97	0	0	5.58	14.63
U-PAR	Urokinase plasminogen activator surface receptor	Q03405	1.86	0	0	6.13	18.69
uPA	Urokinase-type plasminogen activator	P00749	1.09	0	0	5.65	15.43
vWF	von Willebrand factor	P04275	1.09	0	0	11.49	38.74

CV, coefficient of variation; LOD, limit of detection; UniProt ID, universal protein database identification.

[a] All or nearly all values of the control samples, which are requisite to calculate the CVs, were < LOD. If the values of the control samples are < LOD, they are not included in the calculation of the CVs. Therefore, the number of available values was too low to estimate the CV.

Table S3: Biomarker information Inflammation panel

Biomarker ID	Full name	UniProt ID	LOD	Values <LOD (n)	Values <LOD (%)	Intra-Assay CV (%)	Inter-Assay CV (%)
4E-BP1	Eukaryotic translation initiation factor 4E-binding protein 1	Q13541	2.19	0	0	5.78	64.17
ADA	Adenosine Deaminase	P00813	1.06	0	0	6.88	29.35

Biomarker ID	Full name	UniProt ID	LOD	Values <LOD (n)	Values <LOD (%)	Intra-Assay CV (%)	Inter-Assay CV (%)
ARTN	Artemin	Q5T4W7	0.97	1476	93.95	[a]	[a]
AXIN1	Axin-1	O15169	2.24	26	1.65	5.08	48.36
Beta-NGF	Beta-nerve growth factor	P01138	1.68	1549	98.6	[a]	[a]
CASP-8	Caspase-8	Q14790	1.64	215	13.69	6.17	48
CCL11	Eotaxin	P51671	0.75	0	0	5.83	14.16
CCL19	C-C motif chemokine 19	Q99731	1.82	0	0	5.45	15.73
CCL20	C-C motif chemokine 20	P78556	1.64	0	0	7.31	21.3
CCL23	C-C motif chemokine 23	P55773	1.63	0	0	5.38	12.34
CCL25	C-C motif chemokine 25	O15444	0.55	0	0	5.97	10.84
CCL28	C-C motif chemokine 28	Q9NRJ3	0.71	0	0	7.38	12.03
CCL3	C-C motif chemokine 3	P10147	0.14	0	0	6.48	12.74
CCL4	C-C motif chemokine 4	P13236	0.45	0	0	6.06	14.57
CD244	Natural killer cell receptor 2B4	Q9BZW8	2.03	0	0	7.16	21.25
CD40	CD40L receptor	P25942	2.21	0	0	5.33	25.34
CD5	T-cell surface glycoprotein CD5	P06127	0.7	0	0	6.09	20.84
CD6	T cell surface glycoprotein CD6 isoform	P30203	1.5	0	0	11.63	28.56
CD8A	T-cell surface glycoprotein CD8 alpha chain	P01732	1.23	0	0	8.41	14.18
CDCP1	CUB domain-containing protein 1	Q9H5V8	-0.06	0	0	10.33	12.52
CSF-1	Macrophage colony-stimulating factor 1	P09603	1.24	0	0	5.66	8.18
CST5	Cystatin D	P28325	0.57	0	0	4.77	11.88
CX3CL1	Fractalkine	P78423	1.11	0	0	8.39	13.04
CXCL1	C-X-C motif chemokine 1	P09341	2.49	0	0	5.49	42.98
CXCL10	C-X-C motif chemokine 10	P02778	2.98	0	0	5.96	15.71
CXCL11	C-X-C motif chemokine 11	O14625	1.89	0	0	5.59	37.97
CXCL5	C-X-C motif chemokine 5	P42830	3.17	0	0	4.49	40.11
CXCL6	C-X-C motif chemokine 6	P80162	1.28	0	0	5.63	30.23
CXCL9	C-X-C motif chemokine 9	Q07325	1.38	0	0	5.45	12.76

Biomarker ID	Full name	UniProt ID	LOD	Values <LOD (n)	Values <LOD (%)	Intra-Assay CV (%)	Inter-Assay CV (%)
DNER	Delta and Notch-like epidermal growth factor-related receptor	Q8NFT8	1.31	0	0	4.22	9.55
EN-RAGE	Protein S100-A12	P80511	0.13	0	0	7.89	28.18
FGF-19	Fibroblast growth factor 19	O95750	0.89	0	0	5.69	13.34
FGF-21	Fibroblast growth factor 21	Q9NSA1	1.6	0	0	6.26	11.12
FGF-23	Fibroblast growth factor 23	Q9GZV9	2.38	1279	81.41	8.34	9.23
FGF-5	Fibroblast growth factor 5	P12034	0.68	1203	76.58	8.94	8.53
Flt3L	Fms-related tyrosine kinase 3 ligand	P49771	1.89	0	0	6.28	11.58
GDNF	Glial cell line-derived neurotrophic factor	P39905	2.07	721	45.89	8.89	12.11
HGF	Hepatocyte growth factor	P14210	0.93	0	0	5.3	14.82
IFN-gamma	Interferon gamma	P01579	3.08	0	0	6.9	14.08
IL-1 alpha	Interleukin-1 alpha	P01583	-0.62	1493	95.04	[a]	[a]
IL-10RA	Interleukin-10 receptor subunit alpha	Q13651	0.34	512	32.59	8.22	9.7
IL-10RB	Interleukin-10 receptor subunit beta	Q08334	0.73	0	0	6.45	9.64
IL-12B	Interleukin-12 subunit beta	P29460	0.19	0	0	6.62	13.25
IL-15RA	Interleukin-15 receptor subunit alpha	Q13261	0.22	0	0	8.66	9.87
IL-17A	Interleukin-17A	Q16552	1.1	725	46.15	9.99	13.14
IL-17C	Interleukin-17C	Q9P0M4	1.48	878	55.89	6.67	5.76
IL-18R1	Interleukin-18 receptor 1	Q13478	1.36	0	0	5.29	11.1
IL-20	Interleukin-20	Q9NYY1	0.73	1515	96.44	[a]	[a]
IL-20RA	Interleukin-20 receptor subunit alpha	Q9UHF4	1.03	1394	88.73	7.64	9.21
IL-22 RA1	Interleukin-22 receptor subunit alpha-1	Q8N6P7	2.63	1369	87.14	9.04	14.66
IL-24	Interleukin-24	Q13007	1.99	1501	95.54	[a]	[a]
IL-2RB	Interleukin-2 receptor subunit beta	P14784	1.6	1443	91.85	[a]	1.77
IL10	Interleukin-10	P22301	1.99	5	0.32	9.48	16.18
IL13	Interleukin-13	P35225	1.14	1423	90.58	6.96	11.88
IL18	Interleukin-18	Q14116	0.35	0	0	5.62	14.9
IL2	Interleukin-2	P60568	1.48	1568	99.81	[a]	[a]
IL33	Interleukin-33	O95760	1.41	1542	98.15	[a]	[a]

Biomarker ID	Full name	UniProt ID	LOD	Values <LOD (n)	Values <LOD (%)	Intra-Assay CV (%)	Inter-Assay CV (%)
IL4	Interleukin-4	P05112	1.07	1409	89.69	[a]	2.41
IL5	Interleukin-5	P05113	1.34	1415	90.07	8.35	15.58
IL6	Interleukin-6	P05231	1.48	23	1.46	5.27	12.44
IL7	Interleukin-7	P13232	0.96	0	0	7.71	20.38
IL8	Interleukin-8	P10145	0.85	0	0	5.72	13.86
LAP TGF-beta-1	Latency-associated peptide transforming growth factor beta-1	P01137	1.08	0	0	5.26	17.29
LIF	Leukemia inhibitory factor	P15018	0.88	1504	95.74	5.44	7.43
LIF-R	Leukemia inhibitory factor receptor	P42702	0.81	0	0	7.19	11.11
MCP-1	Monocyte chemotactic protein 1	P13500	1.77	0	0	4.94	11.15
MCP-2	Monocyte chemotactic protein 2	P80075	1.93	0	0	8.85	16.51
MCP-3	Monocyte chemotactic protein 3	P80098	1.24	165	10.5	7.67	9.12
MCP-4	Monocyte chemotactic protein 4	Q99616	3.47	0	0	4.77	32.72
MMP-1	Matrix metalloproteinase-1	P03956	1.73	0	0	4.7	28.16
MMP-10	Matrix metalloproteinase-10	P09238	2.43	0	0	4.92	11.4
NRTN	Neurturin	Q99748	1.02	1502	95.61	[a]	[a]
NT-3	Neurotrophin-3	P20783	1.25	6	0.38	7.69	12.76
OPG	Osteoprotegerin	O00300	1.68	0	0	4.57	12.03
OSM	Oncostatin-M	P13725	0.7	0	0	6.52	16.86
PD-L1	Programmed cell death 1 ligand 1	Q9NZQ7	2.82	0	0	5.95	15.45
SCF	Stem cell factor	P21583	1.06	0	0	4.17	8.85
SIRT2	SIR2-like protein 2	Q8IXJ6	4.28	205	13.05	7.04	100.19
SLAMF1	Signaling lymphocytic activation molecule	Q13291	1.49	1007	64.1	[a]	4.39
ST1A1	Sulfotransferase 1A1	P50225	2.67	204	12.99	[a]	[a]
STAMBP	STAM-binding protein	O95630	2.29	0	0	6.65	86.4
TGF-alpha	Transforming growth factor alpha	P01135	0.49	0	0	8.98	10.53
TNF	Tumor necrosis factor	P01375	0.21	0	0	6.08	9.45
TNFB	TNF-beta	P01374	1.21	0	0	7.71	10.28
TNFRSF9	Tumor necrosis factor receptor superfamily member 9	Q07011	1.59	0	0	6.27	10.92

Biomarker ID	Full name	UniProt ID	LOD	Values <LOD (n)	Values <LOD (%)	Intra-Assay CV (%)	Inter-Assay CV (%)
TNFSF14	Tumor necrosis factor ligand superfamily member 14	O43557	1.47	0	0	6.56	35.24
TRAIL	TNF-related apoptosis-inducing ligand	P50591	1.16	0	0	4.94	9.24
TRANCE	TNF-related activation-induced cytokine	O14788	1.02	0	0	8.16	13.39
TSLP	Thymic stromal lymphopoietin	Q969D9	0.64	1476	93.95	[a]	[a]
TWEAK	Tumor necrosis factor (Ligand) superfamily, member 12	O43508	1.67	0	0	6.5	13.86
uPA	Urokinase-type plasminogen activator	P00749	1.69	0	0	4.39	11.53
VEGFA	Vascular endothelial growth factor A	P15692	1.95	0	0	6.67	13.17

CV, coefficient of variation; LOD, limit of detection; UniProt ID, universal protein database identification.

[a] All or nearly all values of the control samples, which are requisite to calculate the CVs, were < LOD. If the values of the control samples are < LOD, they are not included in the calculation of the CVs. Therefore, the number of available values was too low to estimate the CV.

Detailed description concerning the calculations of the outcomes:

Based on the impedance, the BIA generates the parameters resistance and reactance, which were used for the calculations of the variables appendicular skeletal muscle mass (ASMM) and body fat mass index (BFMI). ASMM was calculated using the Sergi equation: ASMM(kg) = -3.964 + 0.227 * resistive index + 0.095 * weight + 1.384 * sex + 0.064 * reactance [1], recommended by the European Working Group on Sarcopenia in Older People in 2019 [2]. Concerning the Sergi equation, the resistive index is the resistance normalized by stature ($height^2$ / resistance). Sex was coded as female = 0 and male = 1. BFMI was calculated using the equation of Kyle et al. [3]. This included first the calculation of fat free mass (FFM) in kg using the formula: FFM = -4.104 + 0.518 * ($height^2$ / resistance) + 0.231 * weight + 0.130 * reactance + 4.229 * sex [4], followed by the calculation of body fat in kg (body fat = weight - FFM) and subsequently the calculation of BFMI (BFMI = body fat / $height^2$).

In the following, we describe the choice of using BIA measurements for our study. Apart from the lower costs, BIA does not expose the participants to radiation as opposed to dual X-ray absorptiometry (DXA) and computed tomography (CT) [5]. This could increase the compliance of the participants and therefore reduce selection bias. Moreover, we specifically used equations to calculate muscle [1] and fat mass [4] for which DXA was used as the reference method. The consensus of the European Working Group on Sarcopenia in Older People from 2019 on which we based our choice to use the Sergi equation for ASMM of BIA measurements, advised the BIA as well as DXA, CT or magnetic resonance imaging (MRI) in research studies to confirm sarcopenia through measuring muscle quantity or quality [2].

Table S4: Definition of the outcomes in the cross-sectional analysis

Outcome variable	Type	Coding	N
ASMM	Continuous (kg)	-	1478
BFMI	Continuous (kg/m^2)	-	1478
Low ASMM[a]	Binary	1: ASMM < 25th sex-specific percentile<br>0: ASMM ≥ 25th sex-specific percentile	1: 370<br>0: 1108
High BFMI[b]	Binary	1: BFMI > 75th sex-specific percentile<br>0: BFMI ≤ 75th sex-specific percentile	1: 370<br>0: 1108
Combination low ASMM[c] & high BFMI[d]	Binary	1: ASMM < 40th sex-specific percentile & BFMI > 60th sex-specific percentile<br>0: Remaining participants	1: 110<br>0: 1368

[a] Cut point for women: 15.26 kg, cut point for men: 21.18 kg

[b] Cut point for women: 13.42 kg/m^2, cut point for men: 9.78 kg/m^2

[c] Cut point for women: 16.08 kg, cut point for men: 22.27 kg

[d] Cut point for women: 12.03 kg/m^2, cut point for men: 8.79 kg/m^2

ASMM, appendicular skeletal muscle mass; BFMI, body fat mass index; N, number of participants

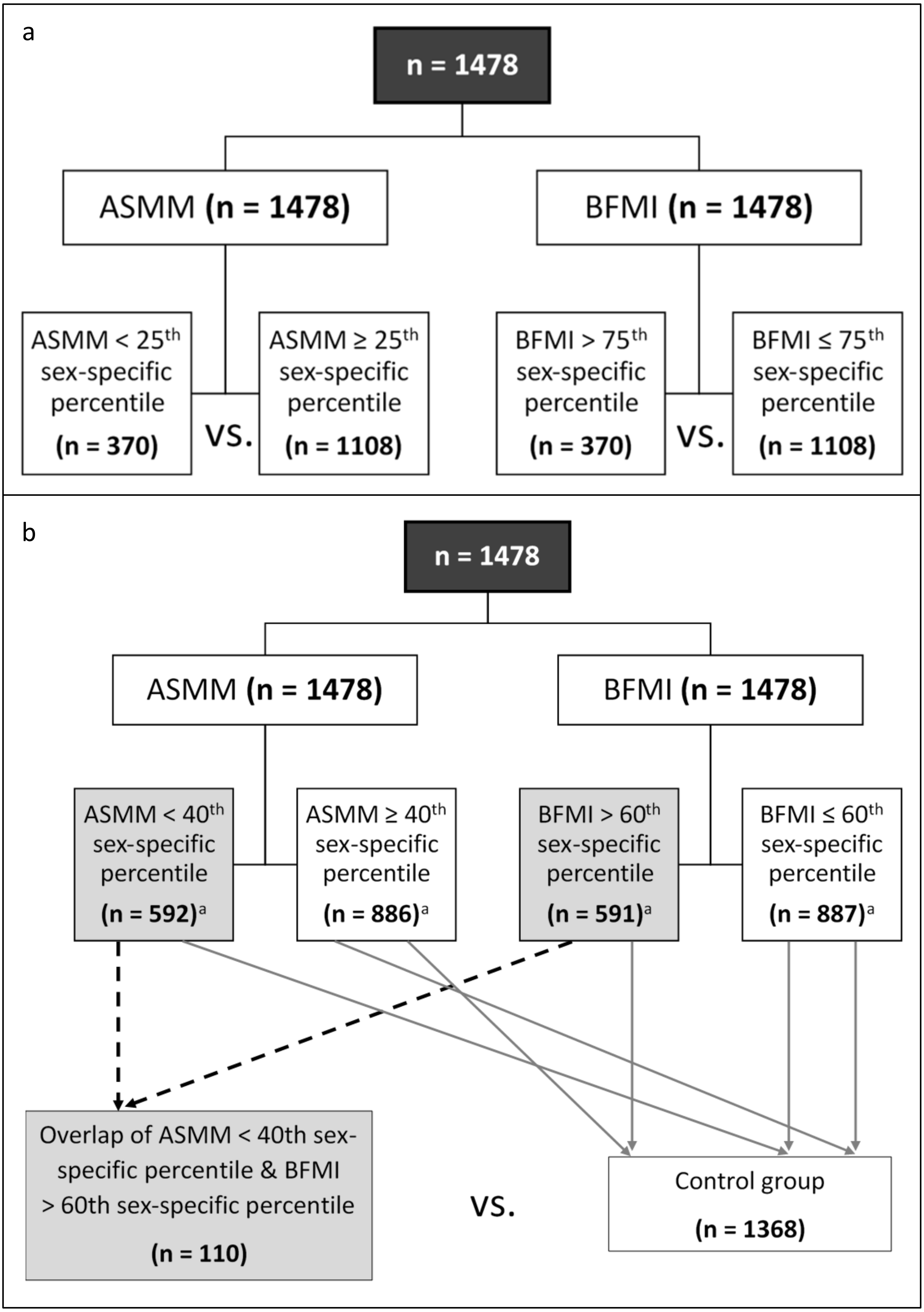

Figure S3: Definition of the outcomes in the cross-sectional analysis

(a) The binary outcome low ASMM consists of the risk group including participants representing the 25 % (n = 370) of participants with the lowest ASMM and its corresponding control group, the remaining 75

% (n = 1108). The binary outcome high BFMI included the 25 % (n = 370) of participants with the highest BFMI and its corresponding control group, the remaining 75 % (n = 1108). (b) The risk group for the combined outcome of low ASMM and high BFMI was determined by intersecting the 40 % of participants with the lowest ASMM and the 40 % of participants with the highest BFMI, illustrated in light grey. This group consists of 7 % (n = 110) of the total study population and the corresponding control group of the remaining participants (n = 1368).

ASMM, appendicular skeletal muscle mass; BFMI, body fat mass index; n, number of participants.

[a] For the group of male participants, one participant had the same value as the cutoff for BFMI. Therefore, the one participant did count into the group of ≤ 60 %. As this was not the case for ASMM, there is one participant less in the group of ASMM ≥ 40 % compared to the group of BFMI ≤ 60 %.

Table S5: Definition of the outcomes in the longitudinal analysis

**Outcome variable**	**Type**	**Coding**	**N**
Relative change in ASMM	Continuous (%)	(follow-up – baseline) / baseline) * 100	608
Relative change in BFMI	Continuous (%)	(follow-up – baseline) / baseline) * 100	608
Strong decrease in ASMM[a]	Binary	1: ASMM relative change < 25th sex-specific percentile<br>0: ASMM relative change ≥ 25th sex-specific percentile	1: 152<br>0: 456
Strong increase in BFMI[b]	Binary	1: BFMI relative change > 75th sex-specific percentile<br>0: BFMI relative change ≤ 75th sex-specific percentile	1: 152<br>0: 456
Combination strong decrease in ASMM[c] & strong increase in BFMI[d]	Binary	1: ASMM relative change < 40th sex-specific percentile &<br>BFMI relative change > 60th sex-specific percentile<br>0: Remaining participants	1: 57<br>0: 551

[a] Cut point for women: -6.81 %, cut point for men: -5.28 %

[b] Cut point for women: 13.19 %, cut point for men: 14.21 %

[c] Cut point for women: -4.63 %, cut point for men: -2.75 %

[d] Cut point for women: 7.78 %, cut point for men: 5.08 %

ASMM, appendicular skeletal muscle mass; BFMI, body fat mass index; N, number of participants.

Detailed description of the covariates:

Albumin was measured in EDTA-plasma with nephelometry using a BN 2 analyzer. Glycated hemoglobin (HbA1c) was analyzed in whole blood with a turbidimetric inhibition immunoassay (TINIA) using a Hitachi 717 (Roche Diagnostics, Mannheim, Germany) [6]. The measurements of high-density lipoprotein (HDL) and triglycerides were described elsewhere [7]. For this analysis, the covariate triglycerides was transformed with natural logarithmic transformation. Estimated glomerular filtration rate (eGFR) was calculated based on measurements of creatinine. Creatinine was measured in serum using enzymatic color test on a Hitachi 917 (Boehringer Mannheim, Mannheim, Germany). The calculations of eGFR with creatinine were based on the publication of Inker et al. in 2012 [8].

The categories of smoking status included never, former or current (at least one cigarette per day) smoker. The definition of the variable physical activity was described elsewhere [9]. The variable education was classified as either > 10 years or ≤ 10 years of education. For the variable alcohol intake, the participants were asked about their consumption of alcoholic beverages on the previous workday and during the previous weekend to estimate the alcohol intake as grams per day. Based on the continuous variable of grams per day, alcohol intake was classified into three categories: men: 0 g/day, 0.1-39.9 g/day, and ≥ 40 g/day; women: 0 g/day, 0.1-19.9 g/day, and ≥ 20 g/day [10]. Blood pressure measurements were described elsewhere [7]. Hypertension was identified if participants had a blood pressure of > 140/90 mmHg or if the participant claimed the intake of antihypertensive medication and was aware of having hypertension [6]. Intake of lipid-lowering medication was defined as intake of at least one medication including Simvastatin, Lovastatin, Pravastatin, Fluvastatin, Atorvastatin, Cerivastatin, Bezafibrat, Gemfirolzil, Fenofibrat, and Etofibrat. Plant-based medication was not included.

Detailed description of the statistical analysis:

All statistical analyses were performed using R, V.3.6.2 [11]. We performed association analysis using the combined method boosting with stability selection [12]. Thereby,

component-wise functional gradient descent boosting of a linear / logistic regression model is combined with the method stability selection, which enables strong control of false positives. We used the R package mboost [13] for boosting and the R package stabs [14] for stability selection. We performed the boosting with an offset encompassing a model including the 13 covariates age, HDL, triglycerides, HbA1c, eGFR, albumin, sex, physical activity, hypertension, smoking status, education, alcohol intake, and intake lipid-lowering medication. As a result, only protein biomarkers that were associated with the outcome independent of the covariates were selected. In a second step, we calculated logistic / linear regression models with the single selected biomarkers adjusted for all 13 covariates and other selected protein biomarkers of the corresponding outcome (model 1). In model 2, we included in addition to model 1 the opponent outcome as a further covariate, i.e. for the outcome ASMM we adjusted for BFMI and vice versa. For all protein biomarkers of which the coefficients became non-significant or changed directions in model 2 compared to model 1, we further included an interaction term of the concerned protein biomarker and the opponent outcome.

The prediction analysis encompassed the calculation of group least absolute shrinkage and selection operator (lasso) using R package grpreg [15] with 100 bootstrap iterations. Based on the 100 lasso calculations in all training samples of the bootstrapping and therefore 100 results concerning the selected variables, we determined the selection frequency of the variables and based on this the final ranking. All variables with the same selection frequency calculated from lasso with bootstrapping have the same rank; e.g. all variables with a selection frequency of 100% have rank 1. Therefore, more than one variable can be assigned to rank 1. We calculated the area under the curve (AUC) of a logistic regression model including 13 classical risk factors (AUC_{basic}) and a model additionally including protein biomarkers (variables of the cross-sectional analysis are listed in Table S8, variables of the longitudinal analysis in Table S11) that were selected in ≥ 90 % of the group lasso bootstrap iterations ($AUC_{extended}$). We additionally calculated their delta AUC ($AUC_{extended}$-AUC_{basic}) to identify the added prediction performance of the most important protein biomarkers on top of the classical risk factors. Therefore, AUCs and delta AUCs were calculated using the R package fbroc [16]. Cross-

validation was used to calculate the arithmetic means of AUCs and delta AUCs over 10 folds. The confidence intervals (CI) of mean AUCs and mean delta AUCs were calculated via 100-fold percentile bootstrapping using the R package boot [17, 18]. Smoothing the ROC curves enabled us to calculate and plot a mean ROC curve illustrated in Figure S4. We smoothed the ROC curve of each of the 10 folds using the function “smooth” from the R package pROC [19] and created the plots of Figure S4 using the R package ggplot2 [20].

As a sensitivity analysis for the prediction analysis, we further compared the results of lasso with bootstrapping with the results of random forest (RF) and support vector machine (SVM). We performed RF using the R package randomForest [21]. R packages caret [22] and e1071 [23] with the “svmlinear2” method were used for SVM with linear Kernel. The ranking of the variables in RF and SVM was according to variable importance measures (VIM), based on the mean decrease in accuracy for categorical outcomes in RF, percentage increase in mean squared error for continuous outcomes in RF, coefficient of determination R^2 for continuous outcomes in SVM and AUC for categorical outcomes in SVM. The top 10 rankings of the most important variables of the lasso with bootstrapping, RF, and SVM were compared in the sensitivity analysis. In all prediction analyses, the classical risk factors and the protein biomarkers were processed equally as possible predictors. Therefore, all variables (13 classical risk factors and 233 protein biomarkers) were available for the ranking.

In the longitudinal analysis, we used the same statistical approach as in the cross-sectional analysis.

Table S6: Baseline (S4) characteristics of the study population

Characteristic	Cross-sectional (n = 1478)	Longitudinal (n = 608)
Age (years) [a]	63.9±5.4	61.9±4.9
Sex male, n (%)	756 (51.2)	315 (51.8)
Triglycerides (mmol/L) [b]	1.41 (1.01)	1.34 (1.02)
HDL cholesterol (mmol/L) [a]	1.49±0.43	1.51±0.43
HbA1c (mmol/mol) [a]	39.5±7.9	38.8±6.8
HbA1c (%)[a]	5.8±0.7	5.7±0.6
Hypertension, n (%)	831 (56.2)	304 (50.0)
eGFR (ml/min/1.73 m^2) [a]	82.4±13.3	84.2±11.7
Albumin (g/L) [a]	38.2±3.9	38.6±4.1
Intake of lipid-lowering medication, n (%)	172 (11.6)	64 (10.5)
Smoking status, n (%)		
Never	710 (48.0)	309 (50.8)
Former	561 (38.0)	231 (38.0)
Current	207 (14.0)	68 (11.2)
Alcohol intake, n (%)		
0 g/day	415 (28.1)	140 (23.0)
Men 0.1–39.9 g/day<br>Women 0.1–19.9 g/day	765 (51.8)	347 (57.1)
Men ≥40 g/day<br>Women ≥20 g/day	298 (20.2)	121 (19.9)
Physical activity, n (%)		
High activity	256 (17.3)	124 (20.4)
Moderate activity	365 (24.7)	170 (28.0)
Low activity	227 (15.4)	96 (15.8)
No activity	630 (42.6)	218 (35.9)
Education ≤ 10 years, n (%)	927 (62.7)	334 (54.9)
ASMM (kg) [a]	19.9±3.9	20.1±4.0
BFMI (kg/m^2) [a]	10.0±3.1	9.5±2.9
Low ASMM, n (%)[c]	370 (25.0)	152 (25.0)
High BFMI, n (%)[d]	370 (25.0)	152 (25.0)
Combination low ASMM and high BFMI, n (%)[e]	110 (7.4)	39 (6.4)

[a] Continuous variables are presented as arithmetic mean±SD.

[b] Natural logarithmic transformed variables are presented as geometric mean (antilog of SE).

[c] 25 % of participants with the lowest ASMM. Cut points were applied for men and women separately.

[d] 25 % of participants with the highest BFMI. Cut points were applied for men and women separately.

[e] Combination of participants, who were categorized in the group of the 40 % of participants with the lowest ASMM and the group of the 40 % of participants with the highest BFMI. Cut points were applied for men and women separately.

ASMM, appendicular skeletal muscle mass; BFMI, body fat mass index; eGFR, estimated glomerular filtration rate; HbA1c, hemoglobin A1c; HDL, high-density lipoprotein.

Table S7: Characteristics of the study population in the longitudinal sample

**Characteristic**	**n = 608**
Variables measured at baseline (S4)	
ASMM (kg) [a]	20.1±4.0
BFMI (kg/m^2) [a]	9.5±2.88
Low ASMM, n (%)[b]	152 (25.0)
High BFMI, n (%)[c]	152 (25.0)
Combination low ASMM and high BFMI, n (%)[d]	39 (6.4)
Variables measured at follow-up (FF4)	
ASMM (kg) [a]	19.7±4.2
BFMI (kg/m^2) [a]	9.7±3.1
Low ASMM, n (%)[b]	152 (25.0)
High BFMI, n (%)[c]	152 (25.0)
Combination low ASMM and high BFMI, n (%)[d]	44 (7.2)
Variables measured at S4 and FF4	
Relative change in ASMM (%) [a]	-2.2±6.6
Relative change in BFMI (%) [a]	3.8±17.1
Strong decrease in ASMM, n (%)[e]	152 (25.0)
Strong increase in BFMI, n (%)[f]	152 (25.0)
Combination of strong decrease in ASMM and strong increase in BFMI, n (%)[g]	57 (9.4)
Strong decrease in ASMM	
Yes (n = 152), relative change in ASMM (%) [a]	-10.1±4.1
No (n = 456), relative change in ASMM (%) [a]	0.4±5.1
Strong increase in BFMI	
Yes (n = 152), relative change in BFMI (%) [a]	25.7±12.2
No (n = 456), relative change in BFMI (%) [a]	-3.5±11.2

[a] Continuous variables are presented as arithmetic mean±SD.

[b] 25 % of participants with the lowest ASMM. Cut points were applied for men and women separately.

[c] 25 % of participants with the highest BFMI. Cut points were applied for men and women separately.

[d] Combination of participants, who were categorized in the group of the 40 % of participants with the lowest ASMM and the group of the 40 % of participants with the highest BFMI. Cut points were applied for men and women separately.

[e] 25 % of participants with the highest decrease in ASMM. Cut points were applied for men and women separately.

[f] 25 % of participants with the highest increase in BFMI. Cut points were applied for men and women separately.

[g] Combination of participants, who were categorized in the group of the 40 % of participants with the highest decrease in ASMM and the group of the 40 % of participants with the highest increase in BFMI. Cut points were applied for men and women separately.

ASMM, appendicular skeletal muscle mass; BFMI, body fat mass index.

Table S8: Cross-sectional analysis – Prediction analysis – Group lasso with 100x bootstrapping

ASMM (kg)		BFMI (kg/m^2)		Low ASMM		High BFMI		Combination low ASMM and high BFMI	
Selected variables	Selection frequency	Selected variables	Selection frequency	Selected variables	Selection frequency	Selected variables	Selection frequency	Selected variables	Selection frequency
Age<br>Sex<br>Physical Activity<br>LEP<br>IGFBP1<br>KLK6<br>MMP2<br>Notch3<br>CXCL9<br>CCL28	100 %	Age<br>Sex<br>eGFR<br>Smoking<br>Education<br>ADM<br>LEP<br>FABP4<br>IGFBP1<br>KLK6<br>CCL4<br>FGF21<br>CCL28	100 %	Age<br>Alcohol<br>IL1RL2<br>PRSS8<br>CCL15<br>Gal4<br>IGFBP1<br>KLK6<br>MB<br>CST5<br>CCL28	100 %	Sex<br>eGFR<br>ADM<br>LEP<br>FABP4<br>IGFBP1	100 %	Age<br>Physical Activity<br>LEP<br>SCGB3A2	100 %
Smoking<br>PRSS27<br>VSIG2<br>DCN<br>IGFBP2<br>TFPI<br>TRAP<br>CST5<br>DNER	99 %	Triglycerides<br>Alcohol<br>PRSS27<br>PSGL1<br>CD8A<br>TWEAK	99 %	SCF<br>LEP<br>SELE<br>TFPI	99 %	Smoking	98 %	Sex<br>MMP2<br>MMP3	99 %
HbA1c<br>eGFR<br>PRSS8<br>CPB1<br>MB	98 %	FGF23<br>XCL1<br>IGFBP7<br>PON3<br>TNFR1<br>MCP1<br>TRAIL	98 %	Physical Activity<br>Intake lipid-lowering medication<br>CD40L<br>VSIG2<br>CXCL10	98 %	Alcohol<br>VEGFA<br>CCL4	97 %	CCL17	98 %
GDF2<br>ALCAM<br>EpCAM<br>CCL4	97 %	SOD2	97 %	CD84<br>SERPINA12<br>GDF15<br>OPN<br>vWF<br>MCP3	97 %	TWEAK	96 %	CCL28	97 %
HGF	96 %	Intake lipid-lowering medication<br>IL4RA<br>IL1RL2<br>GDF2<br>Notch3<br>IFNG<br>MCP2	96 %	eGFR<br>CPB1<br>CTSZ	96 %	Notch3	95 %	MB<br>FGF21	96 %

Alcohol<br>IL7<br>FGF21	95 %	Hyperten-sion<br>TNFRSF-13B<br>CCL16<br>CPB1	95 %	MARCO<br>Notch3	95 %	Age<br>Physical Activity<br>Education<br>PCSK9	94 %	TWEAK	94 %
IL1RL2<br>THBS2<br>XCL1<br>OPN<br>vWF<br>TNFB	94 %	RAGE<br>THBS2	94 %	FABP4	94 %	MMP12<br>VEGFD	93 %	Education	93 %
TRAILR2<br>NT-proBNP	93 %	IL17RA<br>TNFRSF-10C<br>HGF	93 %	RAGE<br>DCN<br>MEPE<br>MMP2<br>PAI<br>IFNG	92 %	Triglycerid-es<br>PRSS27<br>GH	91 %	ENRAGE	92 %
ADM	92 %	CCL15<br>NT-proBNP	92 %	ALCAM<br>LIFR	91 %	TF<br>CD8A<br>PDL1	90 %	MPO	90 %
Intake lipid-lowering medication	91 %	HDL<br>VEGFD<br>PSPD<br>TIMP4<br>TR	91 %	Smoking<br>CD4	90 %				
HDL<br>TNFRSF-11A<br>RAGE<br>CD93<br>CTSZ	90 %	PDL2<br>LDL-RC	90 %						

In the table, only variables are listed that were selected in ≥ 90 times out of 100 group least absolute shrinkage and selection operator bootstrap iterations.

ASMM, appendicular skeletal muscle mass; BFMI, body fat mass index; eGFR, estimated glomerular filtration rate; HbA1c, hemoglobin A1c; HDL, high-density lipoprotein; lasso, least absolute shrinkage and selection operator.

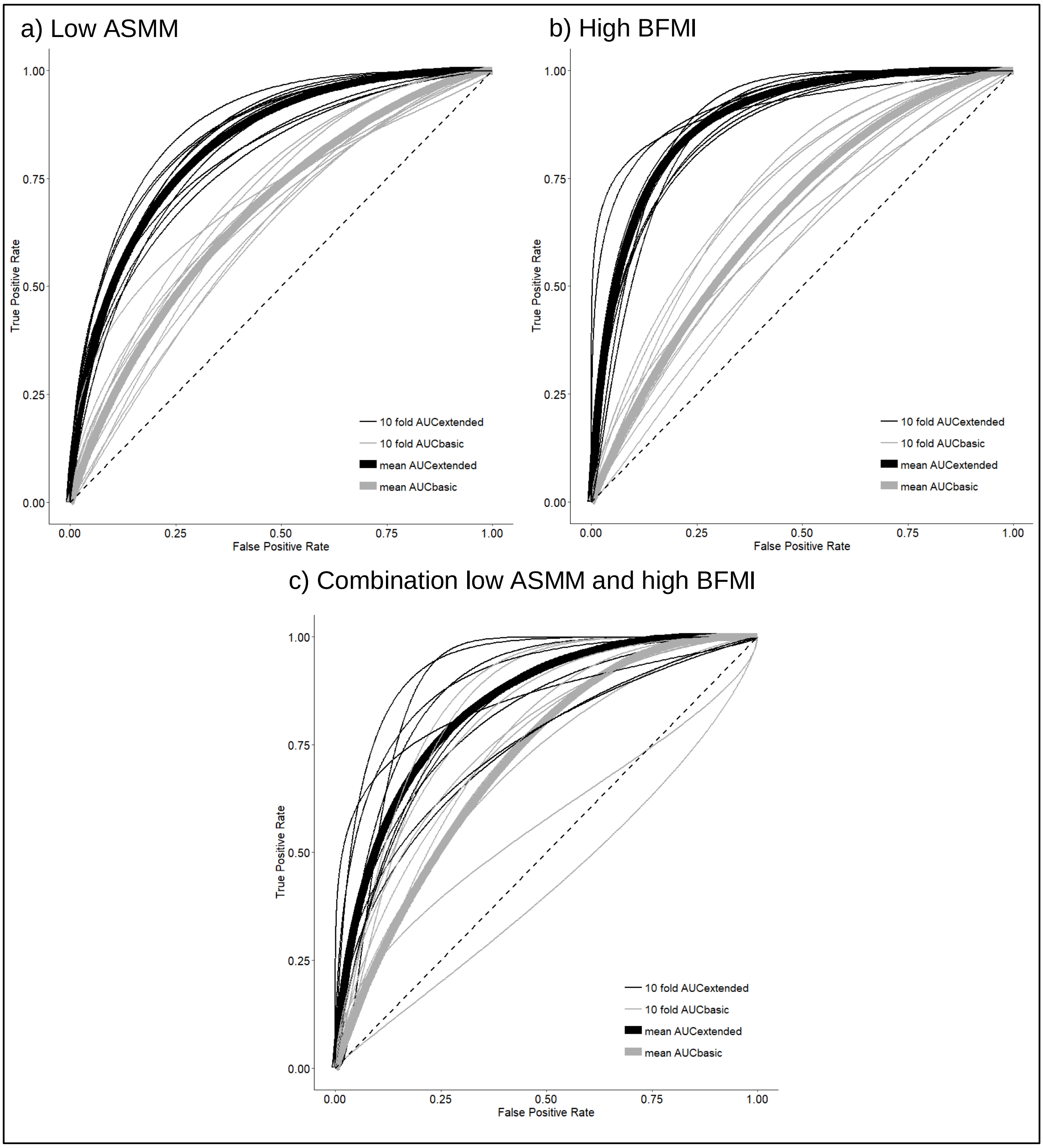

Figure S4: Smoothed ROC curves of 10-fold cross-validation of logistic regression models with classical risk factors (AUC_{basic}) and protein biomarkers in addition to classical risk factors ($AUC_{extended}$)

Smoothed ROC curves of all 10 folds and their mean of the cross-validation are illustrated for the AUCs calculated for a model only including classical risk factors, AUC_{basic} (illustrated in grey), and the AUCs calculated for a model additionally including all protein biomarkers that were selected in ≥ 90 % of the group least absolute shrinkage and selection operator bootstrap iterations, $AUC_{extended}$ (illustrated in black). Bold lines indicate the mean ROC curve of all 10 smoothed ROC curves of the folds, which are

illustrated as thin lines. ROC curves are shown for the outcomes (a) low ASMM, (b) high BFMI, and (c) combination of low ASMM and high BFMI.

AUC_{basic}: AUC of a logistic regression model including 13 classical risk factors (age, high-density lipoprotein, triglycerides, glycated hemoglobin, estimated glomerular filtration rate, albumin, sex, physical activity, hypertension, smoking status, education, alcohol intake, and intake lipid-lowering medication).

$AUC_{extended}$: AUC of the basic model plus all protein biomarkers selected in ≥ 90 % of the group least absolute shrinkage and selection operator bootstrap iterations (variables are listed in Supporting Information, Table S8).

ASMM, appendicular skeletal muscle mass; AUC, area under the curve; BFMI, body fat mass index; ROC, receiver operating characteristic.

Table S9: Cross-sectional analysis – Sensitivity analysis – Comparison of the top 10 most important variables of lasso, random forest, and support vector machine

Rank	Lasso	Random forest	Support vector machine
	ASMM (kg)		
1	Age / Sex / Physical activity / LEP / IGFBP1 / KLK6 / MMP2 / Notch3 / CXCL9 / **CCL28**	Sex	Sex
2		LEP	**LPL**
3		IGFBP1	**GH**
4		**LPL**	HDL
5		**MMP3**	**MMP3**
6		**GH**	GDF2
7		IGFBP2	ACE2
8		**CCL28**	IGFBP1
9		FABP4	**PON3**
10		**PON3**	LEP
	BFMI (kg/m^2)		
1	Age / Sex / eGFR / Education / Smoking / ADM / LEP / FABP4 / **IGFBP1** / KLK6 / CCL4 / FGF21 / CCL28	LEP	LEP
2		FABP4	FABP4
3		Sex	Sex
4		**IGFBP1**	ADM
5		IGFBP2	RARRES2
6		PON3	THBS2
7		ADM	IL1RL2
8		RAGE	**MMP3**

9		GAL9	TNFRSF11A
10		**MMP3**	IL12B
		Low ASMM	
1	**Age** / Alcohol / IL1RL2 / PRSS8 / CCL15 / Gal4 / IGFBP1 / **KLK6** / MB / CST5 / CCL28	**IGFBP2**	**IGFBP2**
2		IGFBP1	IGFBP1
3		**LEP**	**LEP**
4		**PON3**	**PON3**
5		IL1RA	**KLK6**
6		CCL28	**Age**
7		IL27	CCL28
8		CX3CL1	OPN
9		THPO	SCGB3A2
10		CA5A	TFPI
		High BFMI	
1	Sex / eGFR / ADM / LEP / FABP4 / IGFBP1	LEP	LEP
2	Smoking	FABP4	FABP4
3	Alcohol / VEGFA / CCL4	**PON3**	**PON3**
4		ADM	ADM
5		IGFBP1	IGFBP1
6		**IGFBP2**	**IL6**
7		**HGF**	**HGF**
8		**CD163**	THBS2
9		**IL6**	**IGFBP2**
10		TNFRSF11A	**CD163**
		Combination low ASMM and high BFMI	
1	**Age / Physical Activity / LEP /** SCGB3A2	**ADM**	**Age**
2	Sex / MMP2 / MMP3	TFPI	**TIMP4**
3	CCL17	SCF	**Physical activity**
4	CCL28	**GDF15**	**ADM**
5	MB / FGF21	**FABP4**	**GDF15**
6		**TIMP4**	CXCL9
7		PRSS8	IL6
8		CD93	**LEP**
9		AMBP	UPAR
10		KIM1	**FABP4**

Grey shading indicates that the variable was ranked in the top 10 in all three methods (lasso, random forest, and support vector machine); bold print indicates that the variable was ranked in the top 10 in two of the three methods.

All variables with the same selection frequency calculated from lasso with bootstrapping have the same rank; e.g. all variables with a selection frequency of 100% have rank 1. Therefore, more than one variable can be assigned to rank 1.

ASMM, appendicular skeletal muscle mass; BFMI, body fat mass index; eGFR, estimated glomerular filtration rate; lasso, least absolute shrinkage and selection operator.

Results of the longitudinal analysis

Table S10: Association analysis – Boosting with stability selection – Longitudinal analysis

Boosting with stability selection		Linear regression models	
Selected variables	Selection frequency	β (95% CI) (Model 1)	p value
**Relative change in ASMM (%)**			
-			
**Relative change in BFMI (%)**			
CCL4	76%	**-2.29** (-3.72, -0.87)	**0.001700**
ADAMTS13	76%	**-2.22** (-3.55, -0.89)	**0.001123**
CCL15	66 %	**1.92** (0.49, 3.35)	**0.008596**
		**Logistic regression models**	
Selected variables	Selection frequency	OR (95% CI) (Model 1)	p value
**Strong decrease in ASMM**			
NT-proBNP	69 %	**1.40** (1.10, 1.77)	**0.00582**
**Strong increase in BFMI**			
DLK1	65 %	**0.75** (0.60, 0.92)	**0.00681**
**Combination strong decrease in ASMM and strong increase in BFMI**			
NT-proBNP	72 %	**1.60** (1.15, 2.24)	**0.00524**

The cut point for variable selection in the boosting with stability selection was a selection frequency of 63 %, which was determined by the algorithm based on the number of variables available for selection, the number of selected variables per iteration, and the maximum number of tolerable false positives.

Effect estimates have been calculated per 1 SD increase in normalized protein expression values on a log2 scale.

Model 1: Adjustment for all 13 covariates (age, high-density lipoprotein, triglycerides, glycated hemoglobin, estimated glomerular filtration rate, albumin, sex, physical activity, hypertension, smoking status, education, alcohol intake, and intake lipid-lowering medication) as well as all other in the boosting with stability selection selected variables of the corresponding outcome.

Bold print indicates significance.

ASMM, appendicular skeletal muscle mass; BFMI, body fat mass index; β, beta coefficient; CI, confidence interval; OR, odds ratio.

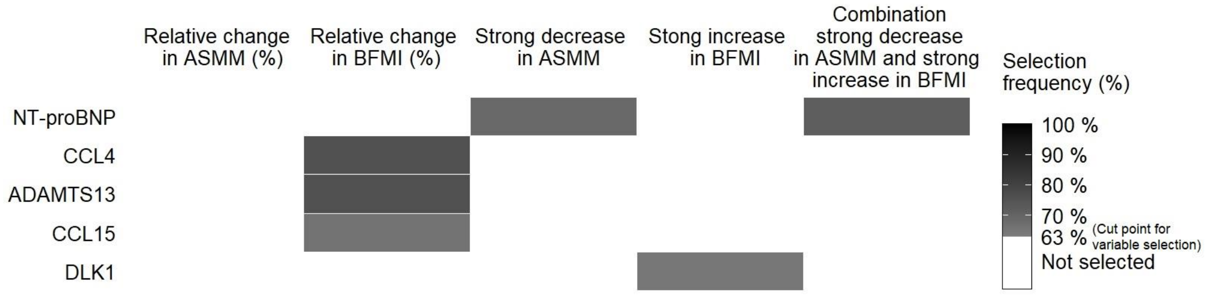

Figure S5: Association analysis – Boosting with stability selection – Comparison of protein biomarker selection between the outcomes – Longitudinal analysis

Protein biomarkers are primarily ordered according to the number of outcomes the biomarkers were selected for and secondary according to their selection for the outcomes in the table from left to right. Only protein biomarkers are included that were selected for at least one outcome. The cut point for variable selection was a selection frequency of 63 %, which was determined by the algorithm based on the number of variables available for selection, the number of selected variables per iteration, and the maximum number of tolerable false positives.

ASMM, appendicular skeletal muscle mass; BFMI, body fat mass index.

Table S11: Prediction analysis – Group lasso with 100x bootstrapping – Longitudinal analysis

Relative change in ASMM (%)		Relative change in BFMI (%)		Strong decrease in ASMM		Strong increase in BFMI		Combination strong decrease in ASMM and strong increase in BFMI	
Selected variables	Selection frequency	Selected variables	Selection frequency	Selected variables	Selection frequency	Selected variables	Selection frequency	Selected variables	Selection frequency
Age	98 %	CCL4	98 %	NT-proBNP	99 %	Age ICAM2	100 %	NT-proBNP	94 %
FAS	95 %	Education Alcohol	96 %	HDL	97 %	PLGR	99 %	PLGR	92 %
FLT3L	90 %	DLK1	95 %			CCL15	96 %		
		ADAMTS-13	93 %			Physical activity	95 %		
		CCL15	92 %			IL6RA	93 %		
		TGM2	90 %			CCL4	92 %		
						CCL16 DLK1	90 %		

In the table, only variables are listed that were selected in ≥ 90 times out of 100 group least absolute shrinkage and selection operator bootstrap iterations.

ASMM, appendicular skeletal muscle mass; BFMI, body fat mass index; HDL, high-density lipoprotein.

Table S12: Prediction analysis – Cross-validated AUCs of logistic regression models with classical risk factors (mean AUC_{basic}) and protein biomarkers in addition to classical risk factors (mean $AUC_{extended}$) – Longitudinal analysis

Outcome	Mean AUC_{basic} (95 % CI)	Mean $AUC_{extended}$ (95 % CI)	Mean delta AUC (95 % CI)
Strong decrease in ASMM	0.54 (0.51, 0.67)	0.57 (0.54, 0.68)	0.03 (0.00, 0.07)
Strong increase in BFMI	0.56 (0.54, 0.68)	0.63 (0.63, 0.75)	0.07 (0.01, 0.11)
Combination strong decrease in ASMM and strong increase in BFMI	0.50 (0.48, 0.70)	0.55 (0.52, 0.72)	0.05 (-0.01, 0.11)

AUC_{basic}: AUC of a logistic regression model including 13 classical risk factors (age, high-density lipoprotein, triglycerides, glycated hemoglobin, estimated glomerular filtration rate, albumin, sex, physical activity, hypertension, smoking status, education, alcohol intake, and intake lipid-lowering medication).

$AUC_{extended}$: AUC of the basic model plus all protein biomarkers selected in ≥ 90 % of the group least absolute shrinkage and selection operator bootstrap iterations (variables are listed in Supporting Information, Table S11).

Delta AUC: $AUC_{extended}$ - AUC_{basic}

AUCs and delta AUCs are arithmetic means of 10-fold cross-validation. The confidence intervals of AUCs and delta AUCs were calculated via 100-fold percentile bootstrapping.

ASMM, appendicular skeletal muscle mass; AUC, area under the curve; BFMI, body fat mass index; CI, confidence interval.

Table S13: Sensitivity Analysis – Comparison of the top 10 most important variables of lasso, random forest, and support vector machine – Longitudinal analysis

Rank	Lasso	Random forest	Support vector machine
		Relative change in ASMM (%)	
1	Age	Age	CA5A
2	FAS	TFPI	Age
3	**FLT3L**	**FLT3L**	CASP8
4	**OPG**	IGFBP2	ALCAM
5	IL6RA	LIFR	IGFBP7
6	CDCP1 / CCL19	CCL23	**OPG**
7	Alcohol / FGF21	IL10RB	HSP27
8	IL12B	GRN	LTBR
9		CSTB	TIE2
10		**IL18R1**	**IL18R1**
		Relative change in BFMI (%)	
1	CCL4	**TPA**	**TPA**
2	Education / Alcohol	CCL4	CCL4
3	**DLK1**	GRN	**LDL-RC**
4	ADAMTS13	**IGFBP2**	IGFBP7
5	**CCL15**	Notch3	Triglycerides
6	TGM2	**CCL15**	**IGFBP2**
7	Smoking	CPB1	**DLK1**
8	CHIT1 / CCL19	MMP9 / HAOX1	FGF21
9		**LDL-RC**	REN
10			AXIN1
		Strong decrease in ASMM	
1	**NT-proBNP**	IGFBP2	**NT-proBNP**
2	HDL	CD40L	HSP27
3	**IL27** / FGF21	**Age**	**vWF**
4	**EGFR**	PCSK9	TNFRSF11A
5	Physical activity	LTBR	**EGFR**
6	RAGE	**TNFR2**	**Age**
7	AGRP / **vWF** / IL12B	TNFRSF10C	RETN
8		ADAMTS13	**IL27**
9		IL17RA / DCN	**TNFR2**
10			GDF15
		Strong increase in BFMI	
1	**Age** / ICAM2	AXIN1	**DLK1**
2	PLGR	NEMO	IGFBP2
3	CCL15	4EBP1	FLT3L
4	Physical activity	PDGFA / MERTK	**IL6RA**
5	**IL6RA**	SIRT2	**Age**
6	**CCL4**	CPB1	LDL-RC
7	CCL16 / **DLK1**	**Triglycerides**	CD163
8	CHIT1	JAMA	CXCL10
9		CXCL16	**CCL4**
10			**Triglycerides** / DCN
		Combination strong decrease in ASMM and strong increase in BFMI	
1	**NT-proBNP**	TIE2	FABP4
2	PLGR	**LEP**	**NT3**
3	**RARRES2**	CXCL9	**LEP**

4	**ADAMTS13**	PCSK9	SCGB3A2
5	**MEPE**	HOSCAR	**NT-proBNP**
6	IL17D	MCP3	**HSP27**
7	**NT3**	CTSZ	**RARRES2**
8	**HSP27**	OPN	**MEPE**
9	IFNG	TNF	IGFBP2
10	MB	IL7	**ADAMTS13**

Grey shading indicates that the variable was ranked in the top 10 in all three methods (lasso, random forest, and support vector machine); bold print indicates that the variable was ranked in the top 10 in two of the three methods.

All variables with the same selection frequency calculated from lasso with bootstrapping have the same rank; e.g. all variables with a selection frequency of 100% have rank 1. Therefore, more than one variable can be assigned to rank 1.

ASMM, appendicular skeletal muscle mass; BFMI, body fat mass index; lasso, least absolute shrinkage and selection operator.

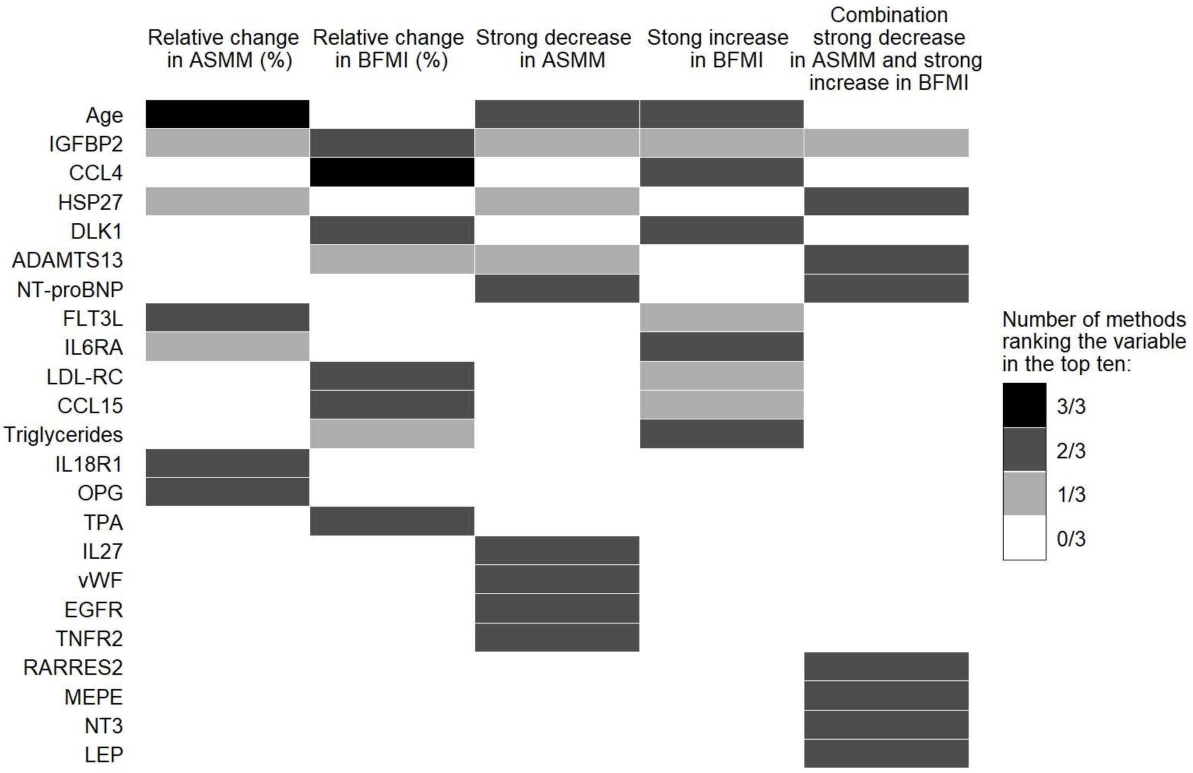

Figure S6: Sensitivity Analysis – Comparison of variables between the outcomes regarding the number of methods that ranked the variables in the top 10 – Longitudinal analysis

Only variables are included that were ranked in the top 10 in at least two of the three analysis methods (group least absolute shrinkage and selection operator with 100x bootstrapping, random forest, and support vector machine) in at least one of the five outcomes. Variables are primarily ordered descending according to the total number (sum of all outcomes) of methods that ranked the variable in the top 10, and secondary according to the outcome in the table from left to right based on the number of methods that ranked the variable in the top 10 for the outcome.

ASMM, appendicular skeletal muscle mass; BFMI, body fat mass index.

References:

1. Sergi G, De Rui M, Veronese N, Bolzetta F, Berton L, Carraro S, et al. Assessing appendicular skeletal muscle mass with bioelectrical impedance analysis in free-living Caucasian older adults. Clin Nutr. 2015;34(4):667-73.
2. Cruz-Jentoft AJ, Bahat G, Bauer J, Boirie Y, Bruyère O, Cederholm T, et al. Sarcopenia: revised European consensus on definition and diagnosis. Age Ageing. 2019;48(1):16-31.
3. Kyle UG, Schutz Y, Dupertuis YM, Pichard C. Body composition interpretation: contributions of the fat-free mass index and the body fat mass index. Nutrition. 2003;19(7-8):597-604.
4. Kyle UG, Genton L, Karsegard L, Slosman DO, Pichard C. Single prediction equation for bioelectrical impedance analysis in adults aged 20–94 years. Nutrition. 2001;17(3):248-53.
5. Marra M, Sammarco R, De Lorenzo A, Iellamo F, Siervo M, Pietrobelli A, et al. Assessment of body composition in health and disease using bioelectrical impedance analysis (BIA) and dual energy X-ray absorptiometry (DXA): a critical overview. Contrast Media Mol Imaging. 2019;2019.
6. Rathmann W, Kowall B, Tamayo T, Giani G, Holle R, Thorand B, et al. Hemoglobin A1c and glucose criteria identify different subjects as having type 2 diabetes in middle-aged and older populations: The KORA S4/F4 Study. Ann Med. 2012;44(2):170-7.
7. Rathmann W, Haastert B, Icks A, Löwel H, Meisinger C, Holle R, et al. High prevalence of undiagnosed diabetes mellitus in Southern Germany: target populations for efficient screening. The KORA survey 2000. Diabetologia. 2003;46(2):182-9.
8. Inker LA, Schmid CH, Tighiouart H, Eckfeldt JH, Feldman HI, Greene T, et al. Estimating glomerular filtration rate from serum creatinine and cystatin C. N Engl J Med. 2012;367(1):20-9.
9. Meisinger C, Löwel H, Thorand B, Döring A. Leisure time physical activity and the risk of type 2 diabetes in men and women from the general population. Diabetologia. 2005;48(1):27-34.
10. Meisinger C, Thorand B, Schneider A, Stieber J, Döring A, Löwel H. Sex differences in risk factors for incident type 2 diabetes mellitus: the MONICA Augsburg cohort study. Arch Intern Med. 2002;162(1):82-9.
11. R Core Team. R: A language and environment for statistical computing. R Foundation for Statistical Computing, Vienna, Austria. URL https://www.R-project.org/. 2019.
12. Hofner B, Boccuto L, Göker M. Controlling false discoveries in high-dimensional situations: boosting with stability selection. BMC Bioinform. 2015;16(1):144.

13. Hothorn T, Buehlmann P, Kneib T, Schmid M, Hofner B. mboost: Model-Based Boosting. R package version 2.9-2. 2020. https://CRAN.R-project.org/package=mboost.
14. Hofner B, Hothorn T. stabs: Stability Selection with Error Control, R package version 0.6-3, https://CRAN.R-project.org/package=stabs. 2017.
15. Breheny P, Huang J. Group descent algorithms for nonconvex penalized linear and logistic regression models with grouped predictors. Stat Comput. 2015;25(2):173-87.
16. Peter E. fbroc: Fast Algorithms to Bootstrap Receiver Operating Characteristics Curves. R package version 0.4.1. https://CRAN.R-project.org/package=fbroc. 2019.
17. Canty A, Ripley B. boot: Bootstrap R (S-Plus) functions. R package version 1.3-25.
18. Davison AC, Hinkley DV. Bootstrap methods and their application: Cambridge: Cambridge University Press; 1997.
19. Robin X, Turck N, Hainard A, Tiberti N, Lisacek F, Sanchez J-C, et al. pROC: an open-source package for R and S+ to analyze and compare ROC curves. BMC Bioinform. 2011;12(1):1-8.
20. Wickham H. ggplot2: Elegant Graphics for Data Analysis. Springer-Verlag New York. 2016.
21. Liaw A, Wiener M. Classification and regression by randomForest. R News. 2002;2(3):18-22.
22. Kuhn M. caret: Classification and Regression Training. R package version 6.0-86. 2020. https://CRAN.R-project.org/package=caret.
23. Meyer D, Dimitriadou E, Hornik K, Weingessel A, Leisch F. e1071: Misc Functions of the Department of Statistics, Probability Theory Group (Formerly: E1071), TU Wien. R package version 1.7-3. 2019. https://CRAN.R-project.org/package=e1071.
